# Risk thresholds for patients to switch between daily tablets and biweekly infusions in second-line treatment for advanced hepatocellular carcinoma: a patient preference study

**DOI:** 10.1186/s12885-022-10388-8

**Published:** 2023-01-19

**Authors:** Neehar D. Parikh, Allicia Girvan, Joshua Coulter, Jonathon Gable, Jiat Ling Poon, Sangmi Kim, Anindya Chatterjee, Marco Boeri

**Affiliations:** 1 Division of Gastroenterology and Hepatology, University of Michigan Health System, Ann Arbor, MI USA; 2 Eli Lilly and Company, Indianapolis, IN USA; 3 RTI Health Solutions, Research Triangle Park, NC USA; 4 RTI Health Solutions, 123B Forsyth House, Cromac Square, Belfast, BT2 8LA UK

**Keywords:** Hepatocellular carcinoma, Patient preferences, Threshold technique, Direct-elicitation

## Abstract

**Background:**

Historically, high hepatocellular carcinoma (HCC)–related mortality has been, in part, due to lack of effective therapies; however, several systemic therapies have been recently approved for HCC treatment, including regorafenib and ramucirumab. These two treatments utilize different routes of administration (four daily tablets and biweekly intravenous infusions, respectively) and have different risks of adverse events (AEs). However, we lack data on patient preferences in balancing the route of administration and risk of AEs in patients with HCC. We aimed to determine patient preferences and trade-offs for second-line treatment in patients with HCC.

**Methods:**

Patients with advanced or metastatic HCC were recruited through their physicians for this study. Patient preferences were assessed by using a modified threshold technique (TT) design in which respondents were asked two direct-elicitation questions before (assuming same safety and efficacy and only varying mode of administration) and after (incorporating the safety profiles of ramucirumab and regorafenib) the TT series on seven risks of clinically relevant AEs.

**Results:**

In total, of the 157 patients recruited by their physicians, 150 were eligible and consented to participate. In the first elicitation question (assuming risk and efficacy were equivalent), 61.3% of patients preferred daily tablets. However, 76.7% of patients preferred the biweekly infusion when the safety profiles of the two available second-line therapies were included. The TT analysis confirmed that preferences for oral administration were not strong enough to balance out the risk of AEs that differentiate the two therapies.

**Discussion:**

We found that when patients were asked to choose between a daily, oral medication and a biweekly IV medication for HCC, they were more likely to choose a daily, oral medication if efficacy and safety profiles were the same. However, when risks of AEs representing the safety profiles of two currently available second-line treatments were introduced in a second direct-elicitation question, respondents often selected an IV administration with a safety profile similar to ramucirumab, rather than oral tablets with a safety profile similar to regorafenib. Our findings indicate that the risk profile of a second-line treatment for HCC may be more important than the mode of administration to patients.

**Supplementary Information:**

The online version contains supplementary material available at 10.1186/s12885-022-10388-8.

## Background

Liver cancer is the third leading cause of cancer deaths worldwide, and hepatocellular carcinoma (HCC) accounts for 75%-85% of cases [1]. Hepatocellular carcinoma is the second deadliest cancer for men and sixth most deadly cancer for women in the world [1, 2]. In the United States (US), HCC is the ninth leading cause of cancer-related deaths [3]. Most HCC develops in the setting of cirrhosis, predominantly caused by viral, metabolic, or alcohol-induced liver disease [3, 4]. Because of inadequate early detection strategies, HCC is often diagnosed at advanced stages, where we lack curative therapies. In 2007, the US Food and Drug Administration (FDA) approved sorafenib, an oral tyrosine kinase inhibitor, as a first-line treatment for HCC. Sorafenib remained the mainstay of HCC therapy, without approved second-line options until, in 2017, the FDA-approved regorafenib, a daily oral tyrosine kinase inhibitor [2]. As of 2021, there are multiple FDA-approved second-line HCC treatments, including cabozantinib, immunotherapy-based regimens, and ramucirumab, a biweekly intravenous (IV) infusion approved in 2019 for patients with a serum alpha-fetoprotein (AFP) level > 400 ng/mL [5–8].

These second-line therapies are characterized by different routes of administration and specific safety profiles, making this a preference-sensitive decision involving patients [9–12]. Preference-sensitive decisions are those in which there are multiple diagnostic or treatment options, none of which is clearly superior over the others, and the option pursued is based on the particular preferences of the decision maker (in our case, the patient). Furthermore, studies suggest that accounting for patient preferences when choosing a therapeutic strategy can improve clinical outcomes [13].

While there have been some studies that examined patient preferences in HCC [14, 15], none have focused on the choice of systemic therapies for HCC. In this study, we aimed to characterize patient preferences when patients are given the option between a profile similar to an oral tyrosine kinase inhibitor (regorafenib) versus a profile similar to an infusional therapy (ramucirumab), with a particular emphasis on the preferences related to mode of administration and risk of side effects.

## Methods

### Study design

We included adult patients (≥ 18 years old) who were able to read English and provide written consent, were diagnosed by a physician with advanced or metastatic HCC, were eligible for systemic therapy, and had progressed on or been intolerant to sorafenib as first-line systemic therapy prior to receiving second-line therapy. To recruit at least 50 patients with elevated baseline AFP (≥ 400 ng/mL) as part of the sample, we required that respondents have a known baseline AFP tumor marker level reported by a physician. Patient preferences were assessed by using a modified threshold technique (TT) [16] administered via an online survey. Respondents were identified and recruited by their primary oncologists. The study was reviewed and deemed exempt by the RTI International institutional review board. The survey was hosted by Global Perspectives and administered between March and October 2020. All patients provided informed consent to participate and received a link to complete the online survey at home or completed the online survey (using a similar link) directly in their physician’s office.

### Survey development

**Fig. 1:**
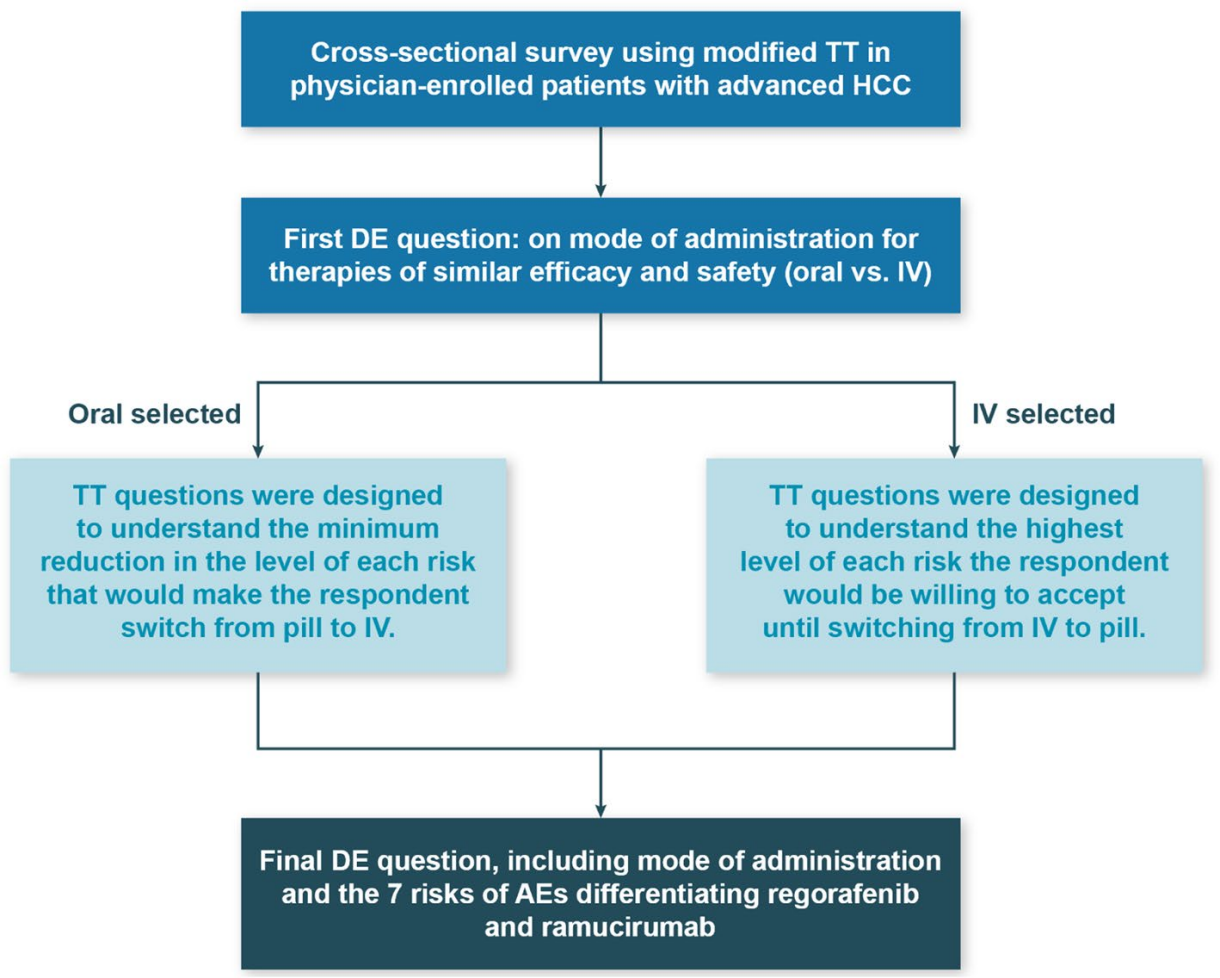
Study design. AE = adverse event; DE = direct elicitation; HCC = hepatocellular carcinoma; IV = intravenous; TT = threshold technique

This study used a modified TT, a stated-preference method used to examine trade-offs patients make when evaluating healthcare decisions, to measure patient preferences around administration and risks (Fig. 1) [16, 17]. Before answering the series of threshold questions, respondents were first presented with a direct-elicitation question asking to choose between two medicines with the same efficacy and safety profiles differentiated only by mode of administration (up to 4 tablets once daily versus an IV infusion once every 2 weeks for 30 to 60 min).

This initial direct-elicitation question was followed by threshold questions developed to investigate seven adverse event (AE) risks that are considered clinically relevant, are the most frequent AEs that impact the daily experience of a patient, have reportedly resulted in treatment dose reduction or discontinuation, and differentiate regorafenib from ramucirumab, as assessed by clinical experts (Table 1) [18–20]. Efficacy and other AEs with similar incidence between regorafenib and ramucirumab were excluded from the list of attributes. The threshold questions asked patients to assume that efficacy and other AEs not included in the choice questions are constant across the alternative options. Each AE risk was presented in a series of threshold questions. Four of the AE risks (risk of hypertension, risk of decreased appetite, risk of diarrhea, and risk of hand-foot skin reaction) favor ramucirumab over regorafenib, while the remaining three (risk of ascites, risk of proteinuria, risk of peripheral edema) favor regorafenib over ramucirumab.

**Table 1:** Survey attributes and profiles for regorafenib and ramucirumab

Attribute	Baseline Profiles, %		Baseline Reference and Target Profiles if Tablets Once Every Day Is Preferred to IV Once Every 2 Weeks, %		Baseline Reference and Target Profiles if Intravenous Infusion Once Every 2 Weeks Is Preferred to Tablets Once Every Day, %	
	Regorafenib	Ramucirumab	Reference Profile (Ramucirumab)	Target Profile (Regorafenib)	Reference Profile (Regorafenib)	Target Profile (Ramucirumab)
Risk of hypertension	25 (31 vs. 6)	11 (24 vs. 13)	25 (31 vs. 6)	11 (24 vs. 13)	11 (24 vs. 13)	25 (31 vs. 6)
Risk of decreased appetite	16 (31 vs. 15)	3 (23 vs. 20)	16 (31 vs. 15)	3 (23 vs. 20)	3 (23 vs. 20)	16 (31 vs. 15)
Risk of hand-foot skin reaction	45 (53 vs. 8)	0 (not reported)	45 (53 vs. 8)	0 (not reported)	0 (not reported)	45 (53 vs. 8)
Risk of diarrhea	26 (41 vs. 15)	1 (16 vs. 15)	26 (41 vs. 15)	1 (16 vs. 15)	1 (16 vs. 15)	26 (41 vs. 15)
Risk of ascites	0 (16 vs. 16)	11 (18 vs. 7)	11 (18 vs. 7)	0 (16 vs. 16)	0 (16 vs. 16)	11 (18 vs. 7)
Risk of proteinuria	0 (not reported)	16 (20 vs. 4)	16 (20 vs. 4)	0 (not reported)	0 (not reported)	16 (20 vs. 4)
Risk of peripheral edema	4 (16 vs. 12)	11 (25 vs. 14)	11 (25 vs. 14)	4 (16 vs. 12)	4 (16 vs. 12)	11 (25 vs. 14)
Mode of administration	Four oral tablets once every day	IV once every 2 weeks for 30 to 60 min	Four oral tablets once every day	IV once every 2 weeks for 30 to 60 min	Four oral tablets once every day	IV once every 2 weeks for 30 to 60 min

Note: The risks for regorafenib and ramucirumab were calculated as differences from the placebo arm in the respective trials. In parentheses, we include values for treatment-related adverse events of any grade for regorafenib (second column) and ramucirumab (third column) vs. placebo

*IV* intravenous infusion

If four oral tablets once every day was selected as the preferred mode of administration in the initial direct-elicitation question, then the treatment profiles in the baseline of the threshold questions were constructed such that the first four risks mirrored regorafenib, while the other three mirrored ramucirumab to complete the TT series. The purpose of this design was to allow all of the improvements to favor the target profile in order to estimate the level of reduction needed in each risk for a respondent to accept an IV infusion once every 2 weeks for 30 to 60 min instead of their preferred option.

Alternatively, if IV infusion was selected as the preferred mode of administration in the initial question, the baseline questions were constructed so that the first four risks mirrored ramucirumab and the last three risks mirrored regorafenib. This design allowed all of the risk increases to be in the target profile in order to estimate the increase in each individual risk the respondent would be willing to accept in order to keep an IV administration once every 2 weeks for 30 to 60 min and not switch to a treatment administered as four oral tablets once every day.

An example TT baseline question is presented in Fig. 2A. The responses to each set of threshold questions were used to define the risk interval for each threshold for each key attribute for each respondent (Fig. 2B and Additional file 1, Figures S1-S13). Following the TT series, respondents were presented with a second direct-elicitation question, which asked them to choose between two treatment profiles that mirrored the regorafenib profile and the ramucirumab profile (Fig. 3).

**Fig. 2:**
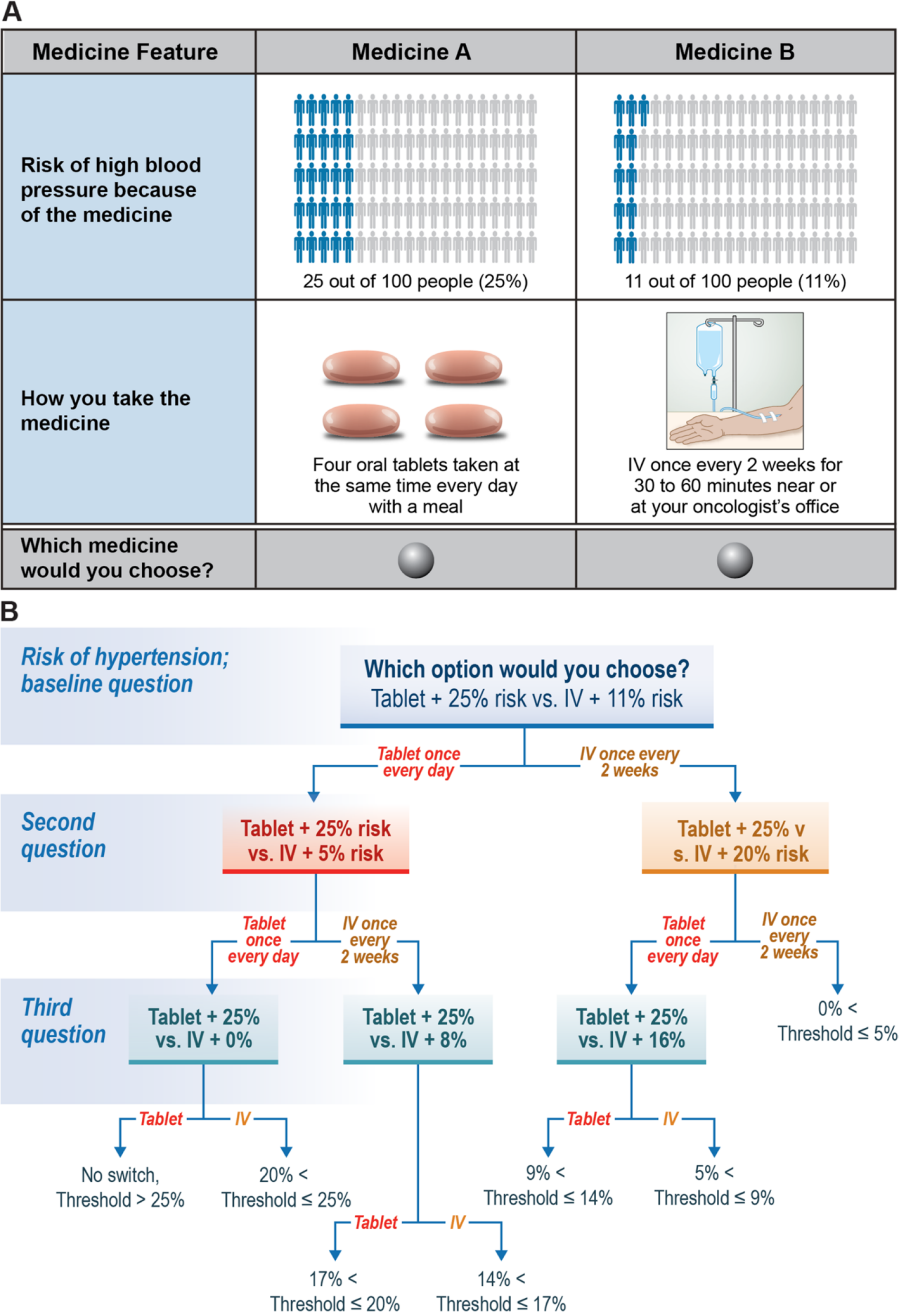
Threshold technique. **A** Example Threshold Technique Question^a^. **B** Example Sequence of Threshold Questions^b^. ^a^Risk of hypertension given as an example of the questions respondents saw during the survey. ^b^Calculations and sequence given for the risk of hypertension if “four tablets” was chosen as the initial preference as an example. All sequences can be found in Additional file 1. IV=intravenous

**Fig. 3:**
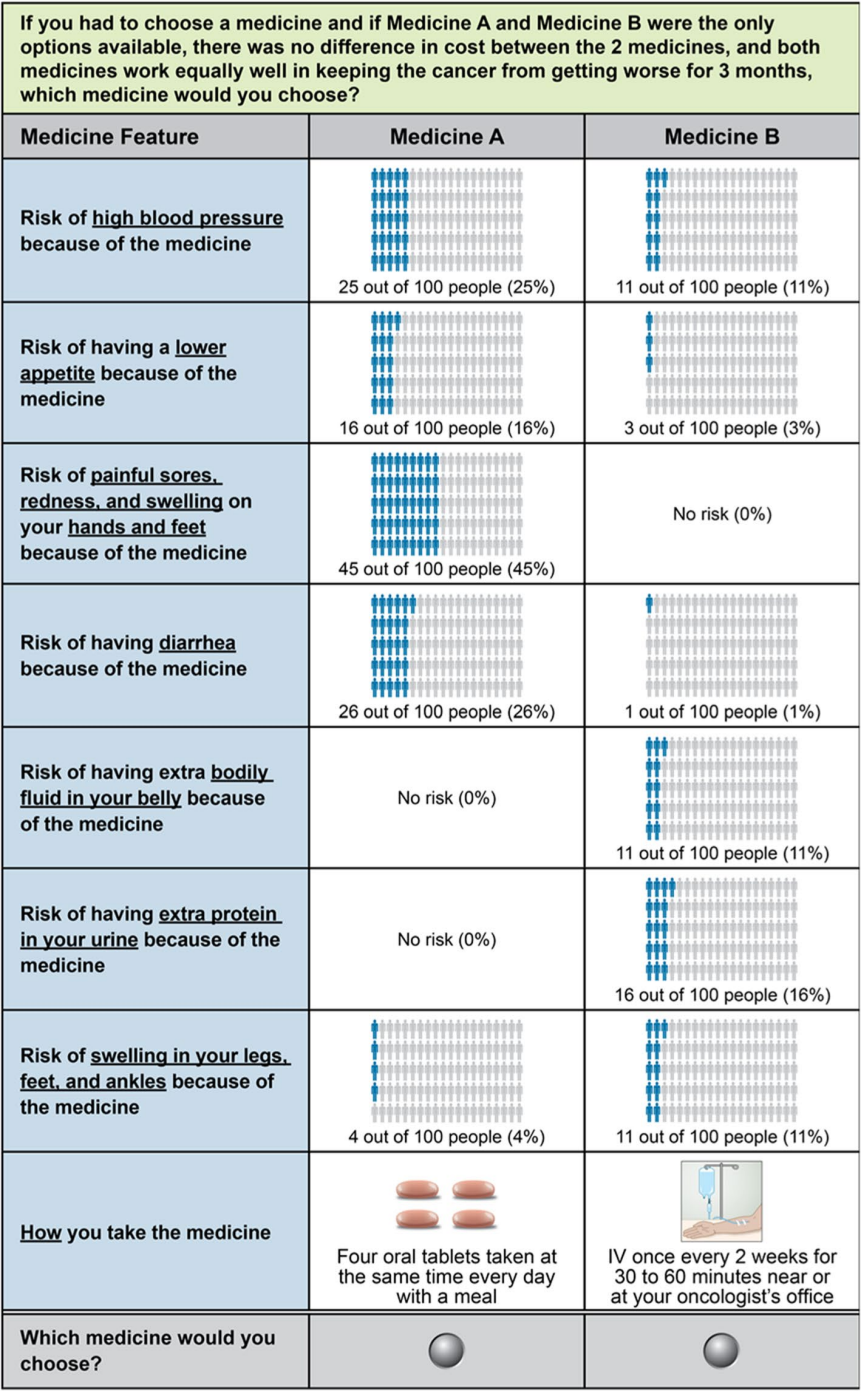
Final direct-elicitation question. IV=intravenous

Pretest interviews were conducted in November 2019 to assess comprehensibility of the survey. Participants in the pretest survey met the same criteria as above, and 9 of 15 had elevated (> 400 ng/mL) AFP. During the pretest interviews, respondents were asked to think aloud as they completed the draft survey. The survey instrument was revised based on observations during pretests, and we repeated the process with the remaining respondents to ensure that the survey instrument was comprehensible to participants and appropriately assessed preferences.

### Statistical analyses

Descriptive statistics were calculated for the overall sample. The data from the two direct-elicitation questions were summarized by using the frequency of response for each profile, and a logistic regression was used to calculate the odds ratio for respondents preferring ramucirumab over regorafenib. The data from the TT series derived from the two modes of administration (depending on whether respondents preferred oral or IV administration in the first direct-elicitation question) were analyzed using two interval regression models. In these regressions, respondents who did not accept any difference in risk in any of the threshold questions have a lower- and upper-bound value equal to 0, which indicated that they were not willing to accept any increased risk. For all respondents in between (e.g., those who accepted increases in risk in some cases but not in others), the lower-bound value is equal to the lowest minimum value the respondent accepted, while the upper-bound value is equal to the lowest minimum value the respondent rejected. The upper- and lower-bound values were then regressed on a constant to obtain the mean acceptable increase in risk across the sample for each risk in isolation. The analysis was repeated for each attribute and mode of administration, resulting in 14 thresholds in total (7 for oral and 7 for IV administration). A covariate-adjusted version of the same regression was used to explore whether and how respondent characteristics (i.e., gender, education, disease state, treatment experience, life orientation) influenced the mean thresholds for IV and oral administrations for each AE risk. Logistic regressions were used to explore whether and how respondent characteristics effected answers to the two direct-elicitation questions. All analyses were performed using STATA, version 16.0 (Statistics/Data Analysis, College Station, Texas).

## Results

### Patient demographics

A total of 157 patients were deemed eligible by their physicians for this study. Of the 157 patients contacted, 150 participants (95.5%) responded to this survey, 54.7% male and 45.3% female. The mean age was 62.1 (range, 34–83) years. Most respondents (58.0%) had been diagnosed with HCC over 1 year earlier, and 87.3% of respondents stated they had metastatic disease at the time they participated in the study. Most respondents (*n* = 124, 82.7%) had elevated physician-reported AFP levels (> 400 ng/mL) and had experience with either IV treatments (73.3%) or three or more daily oral tablets (52.0%). Full demographics can be found in Table 2 and Additional file 1, Table S1, and respondents’ experience with HCC treatment is summarized in Additional file 1, Table S2.

**Table 2:** Demographic characteristics of the respondents

Characteristic	Respondents (*N* = 150)
Gender, n (%)	
Male	82 (54.7)
Female	68 (45.3)
Age (years)	
Mean (SD)	62.1 (9.43)
Min, Max	34, 83
Race or ethnicity, n (%)^ a^	
White	68 (45.3)
Hispanic or Latino	24 (16.0)
Black or African American	24 (16.0)
Native American or American Indian	2 (1.3)
Asian/Pacific Islander	21 (14.0)
Other	7 (4.7)
Prefer not to say	9 (6.0)
Highest level of education completed, (%)	
High school or equivalent (e.g., GED)	11 (7.3)
Some college but no degree	16 (10.7)
Associate degree (2-year college) or technical school	21 (14.0)
4-year college degree (e.g., BA, BS)	46 (30.7)
Some graduate school but no degree	16 (10.7)
Graduate or professional degree (e.g., MBA, MS, MD, PhD)	30 (20.0)
Prefer not to say	10 (6.7)
Residential area, n (%)	
Rural: areas that are not towns or cities; sometimes called the country	23 (15.3)
Suburban: areas where people live within commuting distance of a city	61 (40.7)
Urban: areas where many people live and work close together; sometimes called the city	66 (44.0)

*GED* General Educational Development, *SD* standard deviation

### Responses to the two direct-elicitation questions

**Fig. 4:**
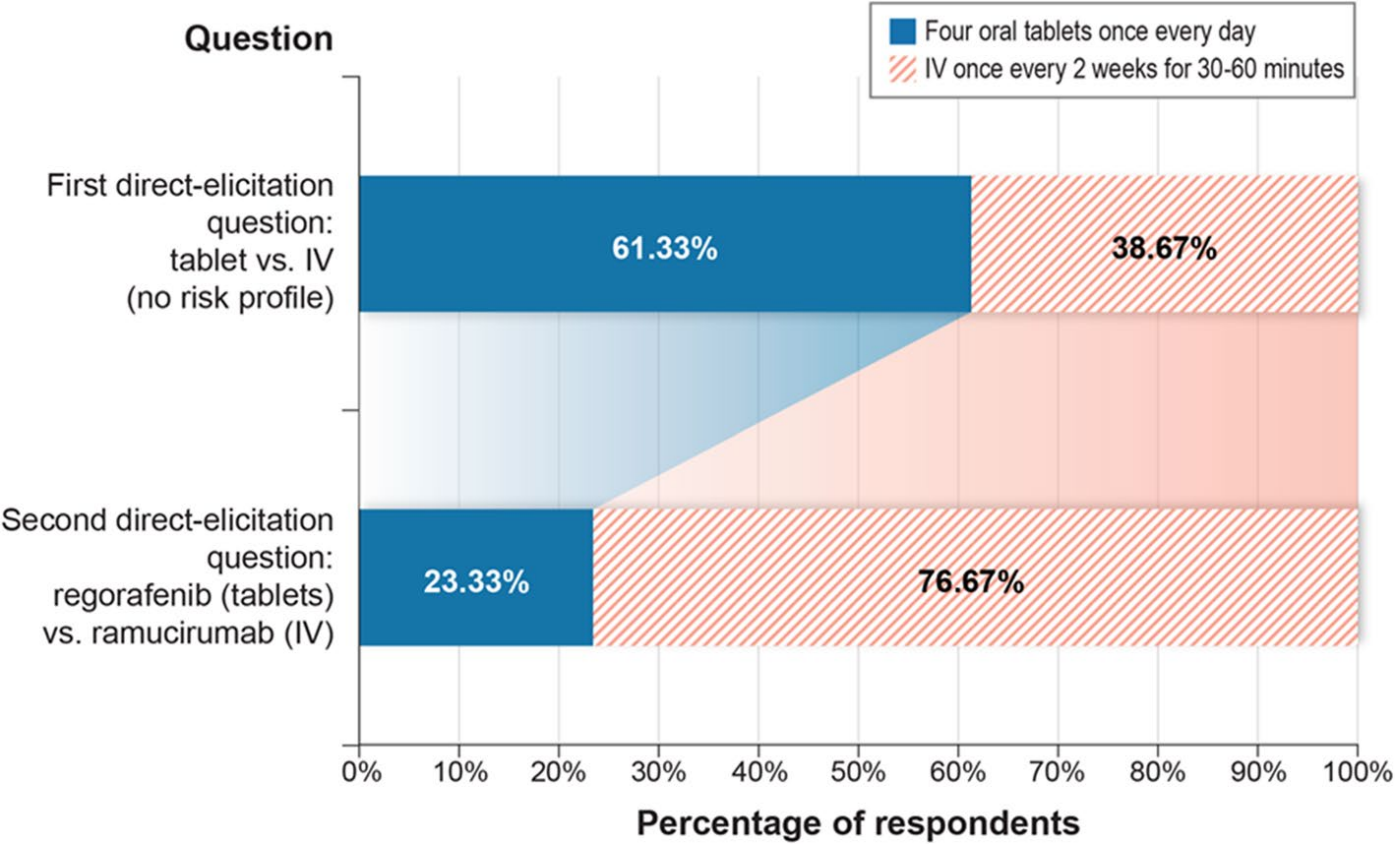
Results from direct-elicitation questions. IV = intravenous

Responses to the two direct-elicitation questions are summarized in Fig. 4. With all else being equal in the first direct-elicitation question, the majority of respondents (*n* = 92, 61.3%) preferred four oral tablets once daily versus IV infusion once every 2 weeks for 30–60 min. However, when all risks were included, a treatment profile similar to IV ramucirumab was preferred by 76.7% of patients, indicating that for many respondents, the utility gain of having oral tablets instead of IV infusion was more than offset by the risks associated with the regorafenib AE profile when compared with ramucirumab (Fig. 4).

### Threshold analysis

**Fig. 5:**
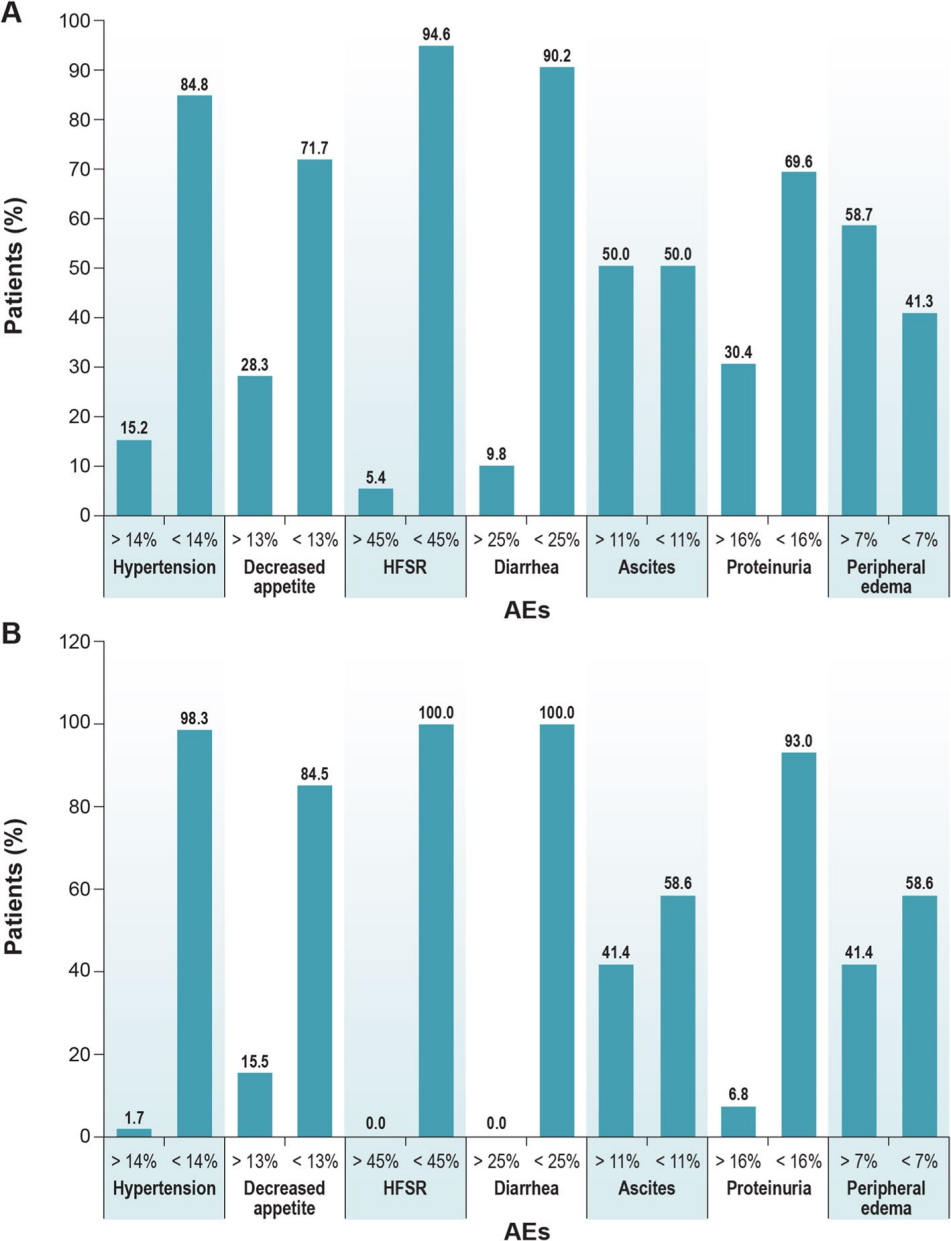
Percentage of respondents on each side of the baseline for each risk. **A** Switch From Oral to IV, *n* = 92. **B** Switch From IV to Oral, *n* = 58. AE = adverse event; HFSR = hand-foot skin reaction; IV = intravenous

The analysis of the TT provides an explanation for respondents switching from tablets to IV infusion when the safety profiles were included in the direct-elicitation questions. To investigate why respondent preferences shifted when risks were included in direct-elicitation questions, we analyzed the TT series for each AE (Additional file 1, Figures S13-S26). Figure 5A presents the percentages of respondents whose minimum reduction in risk of an AE needed to switch from tablets every day to IV infusion every 2 weeks was higher or lower than the baseline reduction in the level of risk. For most AEs, the majority of respondents (69.6% to 94.6%, depending on the risk) had a minimum reduction in risk needed to switch from tablets to IV infusion lower than the baseline reduction, except for ascites (50%) and peripheral edema (41.3%).

Figure 5B presents the percentages of respondents whose maximum acceptable increase in risk of an AE to keep IV administration is higher or lower than the baseline increase in the level of risk. For all the AEs, most respondents (58.6% to 100%) had a maximum increase in risk acceptable to keep IV administration rather than switching to tablets that was lower than the baseline. All respondents in the IV series had a maximum increase in risk acceptable to keep IV administration rather than switching to tablets that was lower than the baseline increase for the risk of diarrhea and hand-foot reaction. As seen in Table 3, for respondents who initially preferred tablets, the risk threshold estimates for which they were indifferent between tablets and IV infusion for hypertension (7.07%), lower appetite (7.94%), hand-foot skin reaction (9.84%), and diarrhea (6.83%) were significantly lower than the baseline risk level. The mean threshold risk estimate for which respondents were indifferent between tablets and IV infusion for peripheral edema was significantly lower than the baseline risk (Table 3). For respondents who initially preferred IV administration, the mean risk threshold estimates for which they were indifferent between tablets and IV infusion for all risks were higher than the baseline risk, indicating that, on average, for each risk independently, respondents would prefer to switch to tablet over IV administration at baseline level.

**Table 3:** Results of constant-only threshold models

Minimum reduction in risk of:	Mean Threshold Estimate	Standard Error	95% CI
**Tablet sample (*n* = 92)**			
Hypertension from 25% needed to switch from tablets to IV (baseline difference = 14%)	7.07	0.97	(5.18–8.97)
Decreased appetite from 16% needed to switch from tablets to IV (baseline difference = 13%)	7.94	0.87	(6.23–9.64)
Hand-foot skin reaction from 45% needed to switch from tablets to IV (baseline difference = 45%)	9.84	1.28	(7.35–12.34)
Diarrhea from 26% needed to switch from tablets to IV (baseline difference = 25%)	6.83	0.84	(5.17–8.48)
Ascites from 11% needed to switch from tablets to IV (baseline difference = 11%)	10.07	1.22	(7.68–12.45)
Proteinuria from 16% needed to switch from tablets to IV (baseline difference = 16%)	8.83	1.13	(6.62–11.05)
Peripheral edema from 11% needed to switch from tablets to IV (baseline difference = 7%)	9.36	1.06	(7.28–11.44)
**Intravenous sample (*n* = 58)**			
Hypertension from 11% needed to keep IV instead of switching to tablets (baseline difference = 14%)	3.36	0.51	(2.35–4.36)
Decreased appetite from 3% needed to keep IV instead of switching to tablets (baseline difference = 13%)	6.92	0.88	(5.19–8.65)
Hand-foot skin reaction from 0% needed to keep IV instead of switching to tablets (baseline difference = 45%)	7.16	0.61	(5.96–8.36)
Diarrhea from 1% needed to keep IV instead of switching to tablets (baseline difference = 25%)	6.66	0.58	(5.52–7.80)
Maximum increase in risk of ascites from 0% needed to keep IV instead of switching to tablets (baseline difference = 11%)	8.51	0.70	(7.15–9.87)
Maximum increase in risk of proteinuria from 0% needed to keep IV instead of switching to tablets (baseline difference = 16%)	8.35	0.81	(6.77–9.94)
Maximum increase in risk of peripheral edema from 4% needed to keep IV instead of switching to tablets (baseline difference = 11%)	5.41	0.57	(4.28–6.54)

*CI* confidence interval, *IV* intravenous

### Heterogeneity exploration

In addition to the analysis at a sample level, preference heterogeneity was explored in both the interval regression model on the thresholds and the logistic regression on the choice data from the direct-elicitation questions. A set of explanatory variables (e.g., gender, education, disease state, treatment experience, life orientation) were used to explore whether and how respondent characteristics influenced the mean threshold for each risk, as well as the probability of selecting a medication in the two direct-elicitation questions (Tables S2 and S3). None of the characteristics included in the IV preference model had a statistically significantly negative effect on mean threshold estimates, holding all other variables constant.

When comparing differences in preferences based on experience with modes of administration, we found that respondents with experience taking three or more oral tablets daily compared with those who did not had a higher minimum reduction of risk of hypertension needed to switch from tablets to IV infusion and a higher maximum increase in risk of ascites, proteinuria, and peripheral edema acceptable to keep IV infusion instead of switching to tablets. Respondents who had taken medicine intravenously had a lower minimum reduction of risk of peripheral edema needed to switch from tablets to IV infusion compared with those who had not received IV medication previously.

Respondents with a 4-year degree or higher more readily switched from tablets to IV infusion when considering the risk of hand-foot skin reaction and diarrhea compared with those without a 4-year degree. Analysis of the population that preferred IV administration found that respondents with metastatic cancer had a higher acceptable risk threshold for hand-foot skin reaction and diarrhea to keep IV administration over switching to tablets.

In the first direct-elicitation question (with mode and frequency of administration being the only difference between the alternatives), respondents who had experience taking three or more oral tablets daily were associated with a statistically significantly lower likelihood of selecting tablets over IV administration compared with those who did not report experience taking three oral tablets daily, holding all other variables constant. In the second direct-elicitation question (in which the two full profiles were compared), respondents who had an AFP level greater than 400 ng/mL were associated with a statistically significantly higher likelihood of selecting a ramucirumab-like profile over a regorafenib-like profile compared with those who had an AFP lower than 400 ng/mL, holding all other variables constant.

## Discussion

Previous studies have shown that cancer patients, across a range of subtypes, would choose to take medication as a daily, oral tablet over any other mode of administration when given the choice, primarily because of the ease and convenience [21–24]. Our study found the same trend, when patients were asked to choose between a daily, oral medication and a biweekly IV medication for treating their HCC, if efficacy and safety profiles were the same. However, when risks of AEs representing the safety profiles of the two currently available second-line treatments were introduced in a second direct-elicitation question, respondents often selected an IV administration with a safety profile similar to ramucirumab, rather than oral tablets with a safety profile similar to regorafenib.

To explain this shift in preferences between the two direct-elicitation questions, the level of each risk of AE included in the profile that exactly offsets the utility gained by having the preferred mode of administration was determined using the TT. Data from the TT show that for all the AE risks, most respondents who preferred oral tablets had a minimum reduction in risk needed to switch from tablets to IV infusion lower than the baseline reduction, except for ascites and peripheral edema. Similarly, when looking at respondents who preferred IV administration, for all the AEs, most respondents had a maximum increase in risk acceptable to keep IV infusion rather than switching to tablets lower than the baseline. All respondents in the IV series had a maximum increase in risk acceptable to keep IV infusion rather than switching to tablets that was lower than the baseline increase for the risk of diarrhea and hand-foot reaction. Similar shifts in preference from oral to IV administration when AEs are considered have been previously reported in patients with breast cancer and those with HCC [14, 21, 23]. A study of patients with HCC that compared preferences among available treatment options sorafenib, lenvatinib, and atezolizumab plus bevacizumab and SIR-Spheres found patients preferred therapies that provided them with lower risk of AEs over therapies that could prolong their lives by a few additional months [25].

Because ramucirumab is specifically indicated for patients with elevated AFP levels, we also examined the preferences of patients based on their AFP levels. As we saw with the main cohort, patients with elevated levels of AFP (> 400 ng/mL) preferred oral administration when the safety profiles were the same, but when risks were introduced, they preferred an IV treatment administered with a safety profile similar to ramucirumab. While this study was not able to discern a reason for this preference, other studies have shown that ramucirumab can prolong overall survival in patients with high AFP levels [4, 7, 26].

A major takeaway from this study and many like it is that maintaining quality of life by reducing AEs and using convenient modes of administration are of a high priority for many patients with HCC [25, 27, 28]. A recent study of patients with HCC indicates that patients are willing to make tradeoffs on convenience of dosing or even survival to avoid AEs and maintain their daily function [28]. Hypertension is consistently found to be an important AE to avoid for patients with HCC [27, 28], which is supported by our finding that the risk threshold estimates for which respondents were indifferent between tablets and IV infusion for hypertension was half the baseline risk, indicating that when considering hypertension as an AE, patients are willing to assume less risk than would be required for them to switch from IV to tablet. Understanding the preferences of patients with HCC with regards to their treatments is vital to providing the best possible medical guidance and healthcare. Recently, initiatives to gather patient perspectives and integrate patient preferences into their care have been gaining traction, including in life-sustaining treatment decisions [27–29]. This study advances the field of preferences of patients with HCC by shedding light on how patients weigh AEs and modes of administration when making healthcare decisions.

A limitation of this study is that patients who participated may have characteristics and preferences that differ from the overall population of patients in the US who have metastatic HCC. Most notably, 45.3% of respondents to this survey were female, but HCC is 2–3 times more common in men than in women [1, 30]. Because this study recruited during the first 6 months of the COVID-19 pandemic, we were unable to exercise much control over the demographics of respondents while ensuring we gathered a sufficient patient sample. The effects of sociodemographic differences in cancer treatment preferences have not been well established, however, so it is unclear what impact this might have had on the results of this study. In addition, patients who chose to respond to the recruitment invitation may have preferences that differ from those who chose not to respond to the recruitment invitation. While studies have indicated that survey-based studies generally agree with population-based studies, the potential for selection bias among this study cohort is possible [31]. However, data collected on patient demographics and clinical characteristics in this study do indicate that we captured data from a diverse sample, with patients from all residential regions, and with all forms of health insurance, methods of traveling to oncologists, and commute times (Additional file 1, Table S1). This study also evaluated only two HCC second-line therapies on the market and cannot, therefore, measure the degree to which additional available options may impact patients’ decisions in choosing a second-line therapy. However, other available tyrosine kinase inhibitors approved for treatment of HCC have similarly high rates of AEs; over two-thirds of patients treated with regorafenib, lenvatinib, or cabozantinib experience grade 3 or higher AEs [32]. Regorafenib and cabozantinib, two second-line tyrosine kinase inhibitors, have very similar efficacy and mode of administration as well [5, 8, 33, 34]. At the time this study was developed, cabozantinib was not available; however, it appears that some of the findings in this study could be applied to cabozantinib as well because of its similarity to regorafenib. While some studies indirectly comparing the efficacy of regorafenib and ramucirumab have been published since our study was conducted [35], of greatest interest to our study was the risk threshold that was acceptable to patients before switching between two drugs. Therefore, our study was designed to hold the efficacy of these two drugs constant throughout the survey questions. Finally, the data collected using the TT are based on responses to hypothetical choice profiles. These choices are intended to simulate possible treatment decisions but do not have the same clinical, financial, or emotional consequences of real-world decisions. Thus, differences can arise between stated and actual choices. We attempted to limit potential hypothetical bias by constructing choice questions that mimicked realistic clinical choices as closely as possible and mapped clearly into clinical evidence.

This study was performed as a hypothetical exercise for patients who may or may not have had experience with the drug administration routes in question. While this study is valuable as an illustration of how patients weigh the risks and benefits of certain medicines and routes of administration, future studies may also wish to examine the satisfaction of patients who are given choices of this nature when determining their own treatments in the clinic. It would also be interesting in future studies to measure the impact of quality of life on treatment preferences.

## Conclusion

All else being equal, respondents preferred four tablets once every day compared with an IV infusion once every 2 weeks for 30 to 60 min; however, when AE risks were introduced, respondents often selected IV administration rather than tablets, showing that preference for oral administration is not strong enough to balance out the risk of AEs that differentiated the oral treatment profile from the IV treatment profile. This finding was confirmed by examining each AE risk individually using the TT. Patient preferences weighing modes of administration and AEs should play a role in the selection of therapies for HCC.

## Supplementary Information

**Additional file 1:** **Table S1.** Summary of Respondents’ Experience with Hepatocellular Carcinoma Treatments. **Table S2.** Results of Covariate-Adjusted Threshold Model: Tablet Sample (*N* = 92). **Table S3.** Results of Covariate-Adjusted Threshold Model: Intravenous Infusion Sample (*N* = 58). **Figure S1.** Threshold Question Sequence for Risk of Decreased Appetite if “Four Tablets” is Initially Preferred. **Figure S2.** Threshold Question Sequence for Risk of Hand-Foot Skin Reaction if “Four Tablets” is Initially Preferred. **Figure S3.** Threshold Question Sequence for Risk of Diarrhea if “Four Tablets” is Initially Preferred. **Figure S4.** Threshold Question Sequence for Risk of Ascites if “Four Tablets” is Initially Preferred. **Figure S5.** Threshold Question Sequence for Risk of Proteinuria if “Four Tablets” is Initially Preferred. **Figure S6.** Threshold Question Sequence for Risk of Peripheral Edema if “Four Tablets” is Initially Preferred. **Figure S7.** Threshold Question Sequence for Risk of Hypertension if Intravenous Infusion is Initially Preferred. **Figure S8.** Threshold Question Sequence for Risk of Decreased Appetite if Intravenous Infusion is Initially Preferred. **Figure S9.** Threshold Question Sequence for Risk of Hand-Foot Skin Reaction if Intravenous Infusion is Initially Preferred. **Figure S10.** Threshold Question Sequence for Risk of Diarrhea if Intravenous Infusion is Initially Preferred. **Figure S11.** Threshold Question Sequence for Risk of Ascites if Intravenous Infusion is Initially Preferred. **Figure S12.** Threshold Question Sequence for Risk of Proteinuria if Intravenous Infusion is Initially Preferred. **Figure S13.** Threshold Question Sequence for Risk of Peripheral Edema if Intravenous Infusion is Initially Preferred. **Figure S14.** Minimum Reduction in Risk of Hypertension to Switch From Tablets Every Day to Intravenous Infusion Every 2 Weeks. **Figure S15.** Minimum Reduction in Risk of Decreased Appetite to Switch From Tablets Every Day to Intravenous Infusion Every 2 Weeks. **Figure S16. **Minimum Reduction in Risk of Hand-Foot Skin Reaction to Switch From Tablets Every Day to Intravenous Infusion Every 2 Weeks. **Figure S17.** Minimum Reduction in Risk of Diarrhea to Switch From Tablets Every Day To Intravenous Infusion Every 2 Weeks. **Figure S18**. Minimum Reduction in Risk of Ascites to Switch From Tablets Every Day to Intravenous Infusion Every 2 Weeks. **Figure S19. **Minimum Reduction in Risk of Proteinuria to Switch From Tablets Every Day to Intravenous Infusion Every 2 Weeks. **Figure S20.** Minimum Reduction in Risk of Peripheral Edema to Switch From Tablets Every Day to Intravenous Infusion Every 2 Weeks. **Figure S21.** Maximum Increase in Risk of Hypertension to Keep Intravenous Infusion Every 2 Weeks Instead of Switching to Tablets Every Day. **Figure S22.** Maximum Increase in Risk of Lower Appetite to Keep Intravenous Infusion Every 2 Weeks Instead of Switching to Tablets Every Day. **Figure S23.** Maximum Increase in Risk of Hand-foot Reaction to Keep Intravenous Infusion Every 2 Weeks Instead of Switching to Tablets Every Day. **Figure S24.** Maximum Increase in Risk of Diarrhea to Keep Intravenous Infusion Every 2 Weeks Instead of Switching to Tablets Every Day. **Figure S25.** Maximum Increase in Risk of Ascites to Keep Intravenous Infusion Every 2 Weeks Instead of Switching to Tablets Every Day. **Figure S26.** Maximum Increase in Risk of Proteinuria to Keep Intravenous Infusion Every 2 Weeks Instead of Switching to Tablets Every Day. **Figure S27.** Maximum Increase in Risk of Peripheral Edema to Keep Intravenous Infusion Every 2 Weeks Instead of Switching to Tablets Every Day.

## Data Availability

The datasets supporting the conclusions of this article are available upon reasonable request. Data are available to request 6 months after the indication studied has been approved in the US and EU and after primary publication acceptance, whichever is later. No expiration date of data requests is currently set once data are made available. Access is provided after a proposal has been approved by an independent review committee identified for this purpose and after receipt of a signed data sharing agreement. Data and documents, including the study protocol, statistical analysis plan, study report, and blank or annotated case report forms, will be provided in a secure data sharing environment. For details on submitting a request, see the instructions provided at www.vivli.org.

## Abbreviations

AE: Adverse event
AFP: Alpha-fetoprotein
CI: Confidence interval
DE: Direct elicitation
FDA: US Food and Drug Administration
GED: General Educational Development
HCC: Hepatocellular carcinoma
HFSR: Hand-foot skin reaction
IV: Intravenous
TT: Threshold technique
SD: Standard deviation
US: United States
