# Supplementary material for: Risk thresholds for patients to switch between daily tablets and biweekly infusions in second-line treatment for advanced hepatocellular carcinoma: a patient preference study
Source: BMC Cancer. 2023 Jan 19;23:66. doi: 10.1186/s12885-022-10388-8 (PMC9851100; doi:10.1186/s12885-022-10388-8)
Supplement: Supplementary file 1 — Additional file 1: Table S1. Summary of Respondents’ Experience with Hepatocellular Carcinoma Treatments. Table S2. Results of Covariate-Adjusted Threshold Model: Tablet Sample (N = 92). Table S3. Results of Covariate-Adjusted Threshold Model: Intravenous Infusion Sample (N = 58). Figure S1. Threshold Question Sequence for Risk of Decreased Appetite if “Four Tablets” is Initially Preferred. Figure S2. Threshold Question Sequence for Risk of Hand-Foot Skin Reaction if “Four Tablets” is Initially Preferred. Figure S3. Threshold Question Sequence for Risk of Diarrhea if “Four Tablets” is Initially Preferred. Figure S4. Threshold Question Sequence for Risk of Ascites if “Four Tablets” is Initially Preferred. Figure S5. Threshold Question Sequence for Risk of Proteinuria if “Four Tablets” is Initially Preferred. Figure S6. Threshold Question Sequence for Risk of Peripheral Edema if “Four Tablets” is Initially Preferred. Figure S7. Threshold Question Sequence for Risk of Hypertension if Intravenous Infusion is Initially Preferred. Figure S8. Threshold Question Sequence for Risk of Decreased Appetite if Intravenous Infusion is Initially Preferred. Figure S9. Threshold Question Sequence for Risk of Hand-Foot Skin Reaction if Intravenous Infusion is Initially Preferred. Figure S10. Threshold Question Sequence for Risk of Diarrhea if Intravenous Infusion is Initially Preferred. Figure S11. Threshold Question Sequence for Risk of Ascites if Intravenous Infusion is Initially Preferred. Figure S12. Threshold Question Sequence for Risk of Proteinuria if Intravenous Infusion is Initially Preferred. Figure S13. Threshold Question Sequence for Risk of Peripheral Edema if Intravenous Infusion is Initially Preferred. Figure S14. Minimum Reduction in Risk of Hypertension to Switch From Tablets Every Day to Intravenous Infusion Every 2 Weeks. Figure S15. Minimum Reduction in Risk of Decreased Appetite to Switch From Tablets Every Day to Intravenous Infusion Every 2 Weeks. Figure S16. Mi [file 12885_2022_10388_MOESM1_ESM.docx]

Risk Thresholds for Patients to Switch Between Daily Tablets and Biweekly Infusions in Second-Line Treatment for Advanced Hepatocellular Carcinoma: a Patient Preference Study

Neehar D. Parikh, MD MS^1^; Allicia Girvan, PhD^2*^; Joshua Coulter, MA^3^; Jonathon Gable, MS^2*^; Jiat Ling Poon, PhD^2^; Sangmi Kim, PhD^2^; Anindya Chatterjee, MS, PhD^2^; Marco Boeri, PhD^4^

^1^Division of Gastroenterology, University of Michigan Health System, University of Michigan, Ann Arbor, MI, United States; ^2^Eli Lilly and Company, Indianapolis, IN, United States; ^3^RTI Health Solutions, Research Triangle Park, NC, United States; ^4^RTI Health Solutions, Belfast, United Kingdom

*Former employee of Eli Lilly and Company

**Corresponding Author:**

Marco Boeri

123B Forsyth House

Cromac Square

Belfast, BT2 8LA,

United Kingdom

Telephone: +44 (0)161 447 6016

Email: [mboeri@rti.org](mailto:mboeri@rti.org)

Running Head: HCC Treatment Preferences Thresholds

Word Count: 3,284

Number of Tables and Figures: 3 tables, 3 figures in the main text; 3 supplementary tables and 27 supplementary figures.

# Supplementary Material

Additional file 1.docx

Table S1. Summary of Respondents’ Experience with Hepatocellular Carcinoma Treatments

Table S2. Results of Covariate-Adjusted Threshold Model: Tablet Sample (N = 92)

Table S3. Results of Covariate-Adjusted Threshold Model: Intravenous Infusion Sample (N = 58)

Figure S1. Threshold Question Sequence for Risk of Decreased Appetite if “Four Tablets” is Initially Preferred

Figure S2. Threshold Question Sequence for Risk of Hand-Foot Skin Reaction if “Four Tablets” is Initially Preferred

Figure S3. Threshold Question Sequence for Risk of Diarrhea if “Four Tablets” is Initially Preferred

Figure S4. Threshold Question Sequence for Risk of Ascites if “Four Tablets” is Initially Preferred

Figure S5. Threshold Question Sequence for Risk of Proteinuria if “Four Tablets” is Initially Preferred

Figure S6. Threshold Question Sequence for Risk of Peripheral Edema if “Four Tablets” is Initially Preferred

Figure S7. Threshold Question Sequence for Risk of Hypertension if Intravenous Infusion is Initially Preferred

Figure S8. Threshold Question Sequence for Risk of Decreased Appetite if Intravenous Infusion is Initially Preferred

Figure S9. Threshold Question Sequence for Risk of Hand-Foot Skin Reaction if Intravenous Infusion is Initially Preferred

Figure S10. Threshold Question Sequence for Risk of Diarrhea if Intravenous Infusion is Initially Preferred

Figure S11. Threshold Question Sequence for Risk of Ascites if Intravenous Infusion is Initially Preferred

Figure S12. Threshold Question Sequence for Risk of Proteinuria if Intravenous Infusion is Initially Preferred

Figure S13. Threshold Question Sequence for Risk of Peripheral Edema if Intravenous Infusion is Initially Preferred

Figure S14. Minimum Reduction in Risk of Hypertension to Switch From Tablets Every Day to Intravenous Infusion Every 2 Weeks

Figure S15. Minimum Reduction in Risk of Decreased Appetite to Switch From Tablets Every Day to Intravenous Infusion Every 2 Weeks

Figure S16. Minimum Reduction in Risk of Hand-Foot Skin Reaction to Switch From Tablets Every Day to Intravenous Infusion Every 2 Weeks

Figure S17. Minimum Reduction in Risk of Diarrhea to Switch From Tablets Every Day To Intravenous Infusion Every 2 Weeks

Figure S18. Minimum Reduction in Risk of Ascites to Switch From Tablets Every Day to Intravenous Infusion Every 2 Weeks

Figure S19. Minimum Reduction in Risk of Proteinuria to Switch From Tablets Every Day to Intravenous Infusion Every 2 Weeks

Figure S20. Minimum Reduction in Risk of Peripheral Edema to Switch From Tablets Every Day to Intravenous Infusion Every 2 Weeks

Figure S21. Maximum Increase in Risk of Hypertension to Keep Intravenous Infusion Every 2 Weeks Instead of Switching to Tablets Every Day

Figure S22. Maximum Increase in Risk of Lower Appetite to Keep Intravenous Infusion Every 2 Weeks Instead of Switching to Tablets Every Day

Figure S23. Maximum Increase in Risk of Hand-foot Reaction to Keep Intravenous Infusion Every 2 Weeks Instead of Switching to Tablets Every Day

Figure S24. Maximum Increase in Risk of Diarrhea to Keep Intravenous Infusion Every 2 Weeks Instead of Switching to Tablets Every Day

Figure S25. Maximum Increase in Risk of Ascites to Keep Intravenous Infusion Every 2 Weeks Instead of Switching to Tablets Every Day

Figure S26. Maximum Increase in Risk of Proteinuria to Keep Intravenous Infusion Every 2 Weeks Instead of Switching to Tablets Every Day

Figure S27. Maximum Increase in Risk of Peripheral Edema to Keep Intravenous Infusion Every 2 Weeks Instead of Switching to Tablets Every Day

Table S1. Summary of Respondents’ Experience with Hepatocellular Carcinoma Treatments

| Question | Respondents (N = 150) | |
| --- | --- | --- |
| **Physician-reported AFP** | | |
| Mean (SD) | 758.3 (364.82) | |
| Median | 700 | |
| Q1, Q3 | 466, 951 | |
| Min, max | 189, 2139 | |
| **Time since cancer diagnosis** | | |
| Less than 6 months | 30 (20.0%) | |
| 6 months to less than 1 year | 31 (20.7%) | |
| 1 year to less than 2 years | 34 (22.7%) | |
| 2 years to less than 5 years | 39 (26.0%) | |
| More than 5 years | 14 (9.3%) | |
| Don’t know or not sure | 2 (1.3%) | |
| **Presence of metastases** | | |
| Yes | 131 (87.3%) | |
| No | 18 (12.0%) | |
| Don’t know or not sure | 1 (0.7%) | |
| **Experience with medication requiring 3 or more oral tablets a day** | | |
| Yes | | 78 (52.0%) |
| No | | 72 (48.0%) |
| **Experience with medication given as an intravenous infusion (IV)** | | |
| Yes | | 110 (73.3%) |
| No | | 39 (26.0%) |
| Don’t know or not sure | | 1 (0.7%) |

AFP = alpha-fetoprotein; SD = standard deviation.

^a^ Respondents could provide multiple responses to these questions. Therefore, the totals may exceed the number of respondents.

Table S2. Results of Covariate-Adjusted Threshold Model: Tablet Sample (N = 92)

| Variable | Minimum Reduction in Risk of | | | | | | |
| --- | --- | --- | --- | --- | --- | --- | --- |
|  | Hypertension From 25% Needed to Switch from Tablets to IV | Having a Lower Appetite From 16% Needed to Switch from Tablets to IV | Hand-Foot Skin Reaction From 45% Needed to Switch from Tablets to IV | Having Diarrhea From 26% Needed to Switch from Tablets to IV | Ascites from 11% Needed to Switch from Tablets to IV | Proteinuria from 16% Needed to Switch from Tablets to IV | Peripheral Edema from 11% Needed to Switch from Tablets to IV |
|  | Coefficient (SE) | Coefficient (SE) | Coefficient (SE) | Coefficient (SE) | Coefficient (SE) | Coefficient (SE) | Coefficient (SE) |
| Female | 2.61 (1.80) | 0.76 (1.62) | 2.95 (2.76) | 1.02 (1.67) | 2.72 (2.00) | −0.39 (1.99) | 0.39 (1.78) |
| Married | −0.04 (2.13) | 1.37 (1.84) | 0.03 (3.36) | −0.41 (2.00) | 0.15 (2.12) | 1.04 (2.10) | 0.85 (1.92) |
| White | 1.58 (1.75) | −1.24 (1.57) | −0.86 (2.38) | 0.45 (1.66) | −0.94 (1.89) | 0.55 (1.87) | −0.41 (1.67) |
| ≥4 y of college | −3.67 (2.05) | −3.00 (1.92) | −6.29 (3.20)* | −4.36 (1.99)* | 0.20 (1.99) | −3.30 (2.24) | −1.06 (1.92) |
| Retired | −0.79 (1.91) | 0.11 (1.71) | −1.04 (2.53) | 0.13 (1.57) | 1.76 (2.04) | 1.35 (2.14) | 1.99 (1.92) |
| Metastatic disease | 2.22 (1.57) | 1.18 (1.95) | 3.38 (2.26) | 1.20 (1.44) | −3.61 (3.53) | 3.40 (2.28) | 1.24 (2.75) |
| At least 1 h from oncologist’s office | 1.24 (2.16) | 1.89 (2.06) | 0.58 (3.35) | 2.02 (2.22) | 2.31 (2.61) | −0.03 (2.42) | 2.11 (2.45) |
| Experience taking ≥3 tablets | 4.72 (2.07)* | 2.01 (1.76) | 1.25 (2.96) | 2.60 (1.87) | 3.79 (2.07) | 2.31 (2.06) | 2.49 (1.82) |
| Experience with IV administration | 0.14 (2.24) | 0.86 (1.78) | −0.87 (3.31) | 0.25 (2.17) | −0.67 (2.38) | −0.34 (2.30) | −3.92 (2.08)* |
| AFP > 400 ng/mL | 1.06 (2.40) | 1.32 (2.38) | −1.17 (4.22) | 0.86 (2.46) | −4.92 (3.01) | −1.92 (2.81) | −3.16 (2.60) |
| Constant | 2.67 (2.93) | 5.25 (2.65)* | 11.49 (4.15)** | 5.55 (2.56)* | 13.97 (4.13)** | 7.37 (3.60)* | 11.52 (3.64)** |

* Statistically significant at the 95% level; ** statistically significant at the 99% level.

AFP = alpha-fetoprotein; IV = intravenous infusion; SE = standard error.

Note: Robust standard errors are used.

Table S3. Results of Covariate-Adjusted Threshold Model: Intravenous Infusion Sample (N = 58)

| Variable | Maximum Increase in Risk of | | | | | | |
| --- | --- | --- | --- | --- | --- | --- | --- |
|  | Hypertension From 11% Needed to Switch from IV to Tablets | Having a Lower Appetite From 3% Needed to Switch from IV to Tablets | Hand-Foot Skin Reaction From 0% Needed to Switch from IV to Tablets | Having Diarrhea From 1% Needed to Switch from IV to Tablets | Ascites from 0% Needed to Switch from IV to Tablets | Proteinuria from 0% Needed to Switch from IV to Tablets | Peripheral Edema from 4% Needed to Switch from IV to Tablets |
|  | Coefficient (SE) | Coefficient (SE) | Coefficient (SE) | Coefficient (SE) | Coefficient (SE) | Coefficient (SE) | Coefficient (SE) |
| Female | −0.25 (0.66) | −2.64 (1.78) | 1.96 (1.24) | 0.63 (1.17) | 0.35 (1.43) | 0.00 (1.45) | 1.40 (1.17) |
| Married | 1.09 (0.87) | 0.36 (1.68) | 0.82 (1.12) | 0.95 (1.05) | 0.98 (1.28) | 1.99 (1.33) | 2.00 (1.03) |
| White | 2.07 (1.30) | −0.89 (1.85) | 0.80 (1.31) | −1.66 (0.96) | −2.30 (1.44) | 0.16 (1.73) | −0.18 (1.08) |
| ≥4 y college | 0.43 (0.72) | −0.11 (1.62) | 0.75 (1.24) | 1.31 (1.26) | 1.90 (1.46) | 0.72 (1.49) | −0.49 (1.12) |
| Retired | −0.95 (0.93) | −0.22 (1.65) | −0.13 (1.32) | −0.07 (1.04) | 0.50 (1.40) | −0.42 (1.53) | 0.39 (1.08) |
| Metastatic disease | 1.42 (0.80) | 2.72 (1.44) | 2.78 (1.29)* | 2.92 (1.24)* | 2.39 (1.68) | 2.71 (1.59) | 0.63 (1.54) |
| At least 1 h from oncologist’s office | −1.37 (1.20) | −0.69 (1.69) | 1.04 (1.20) | −0.36 (1.00) | −0.44 (1.40) | 0.58 (1.52) | −0.04 (1.01) |
| Experience taking ≥3 tablets | 0.33 (0.87) | 2.06 (1.57) | 2.15 (1.18) | 1.18 (1.22) | 2.85 (1.35)* | 4.01 (1.42)** | 2.47 (1.08)* |
| Experience with IV administration | −0.81 (0.90) | 1.14 (1.57) | −1.24 (1.27) | −1.27 (1.36) | 0.11 (1.53) | 0.02 (1.63) | −0.42 (1.33) |
| AFP > 400 ng/mL | −2.97 (2.46) | −1.54 (3.13) | −2.07 (1.79) | 0.54 (1.24) | −0.33 (1.98) | −0.87 (2.88) | −1.85 (1.53) |
| Constant | 4.21 (2.05)* | 5.51 (3.09) | 3.43 (2.36) | 3.05 (2.19) | 3.81 (2.58) | 2.47 (2.74) | 3.47 (1.92) |

* Statistically significant at the 95% level; ** statistically significant at the 99% level.

AFP = alpha-fetoprotein; IV = intravenous infusion; SE = standard error.

Note: Robust standard errors are used.

Figure S1. Threshold Question Sequence for Risk of Decreased Appetite if “Four Tablets” is Initially Preferred


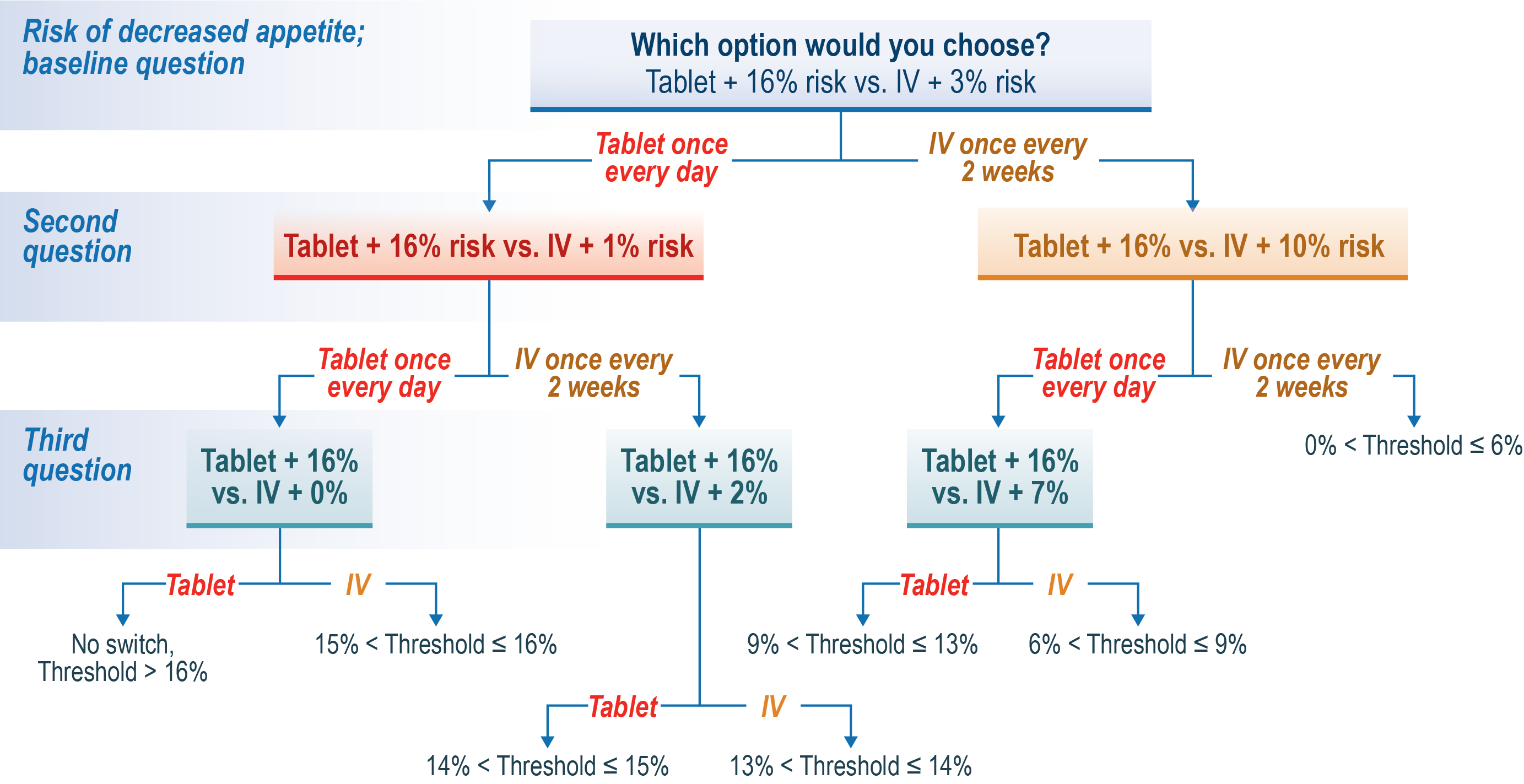


Figure S2. Threshold Question Sequence for Risk of Hand-Foot Skin Reaction if “Four Tablets” is Initially Preferred


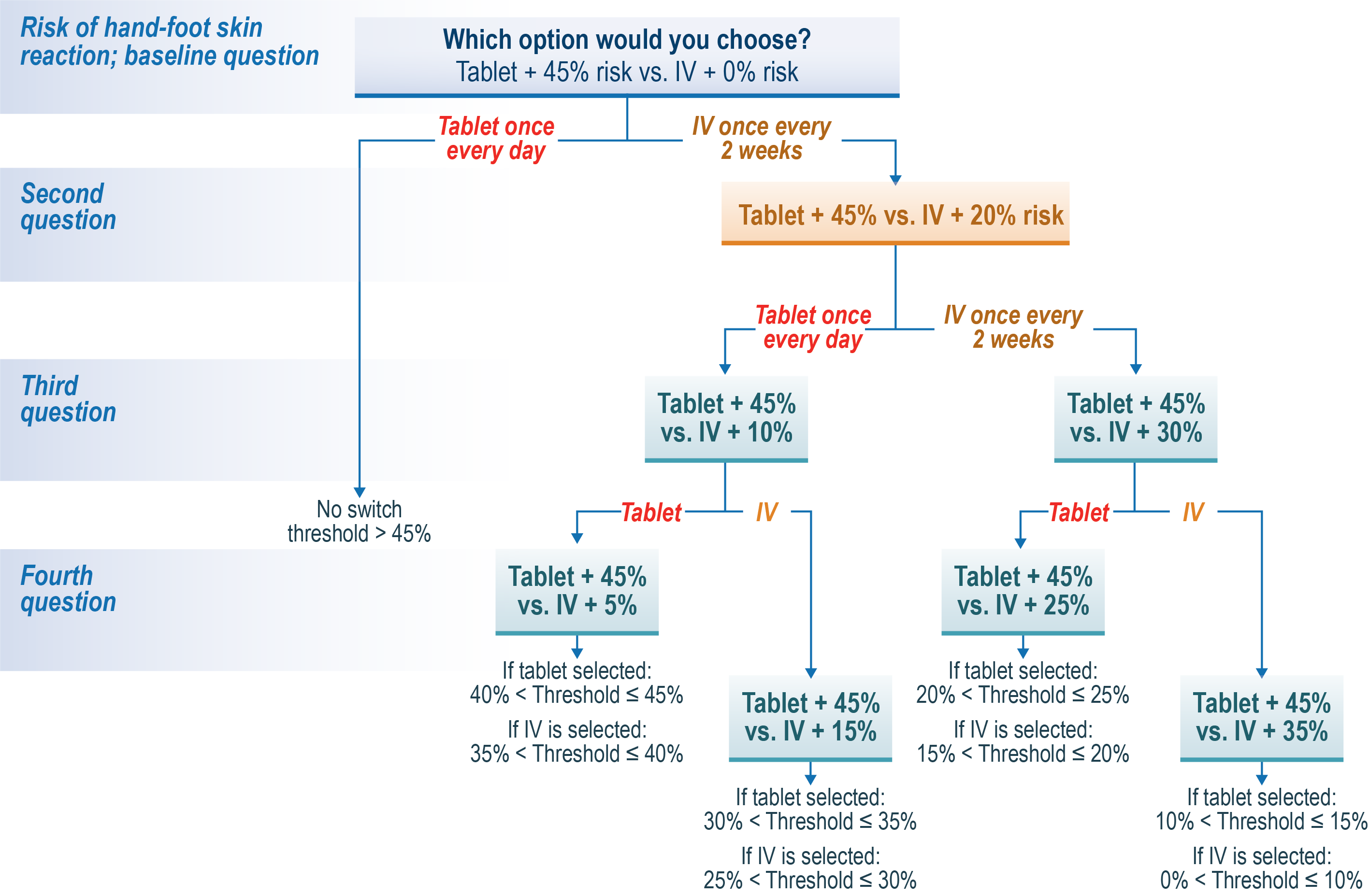


Figure S3. Threshold Question Sequence for Risk of Diarrhea if “Four Tablets” is Initially Preferred


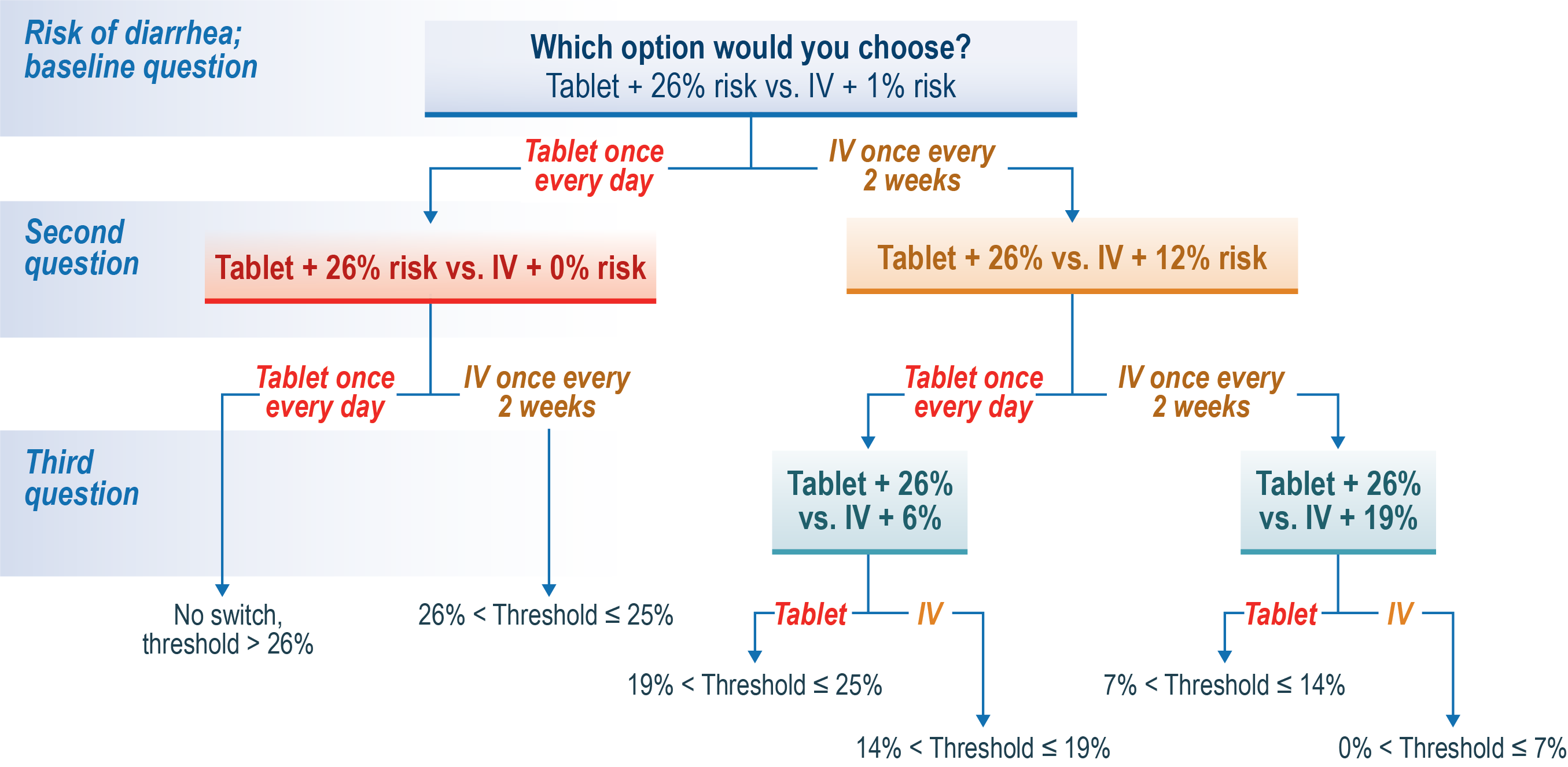


Figure S4. Threshold Question Sequence for Risk of Ascites if “Four Tablets” is Initially Preferred


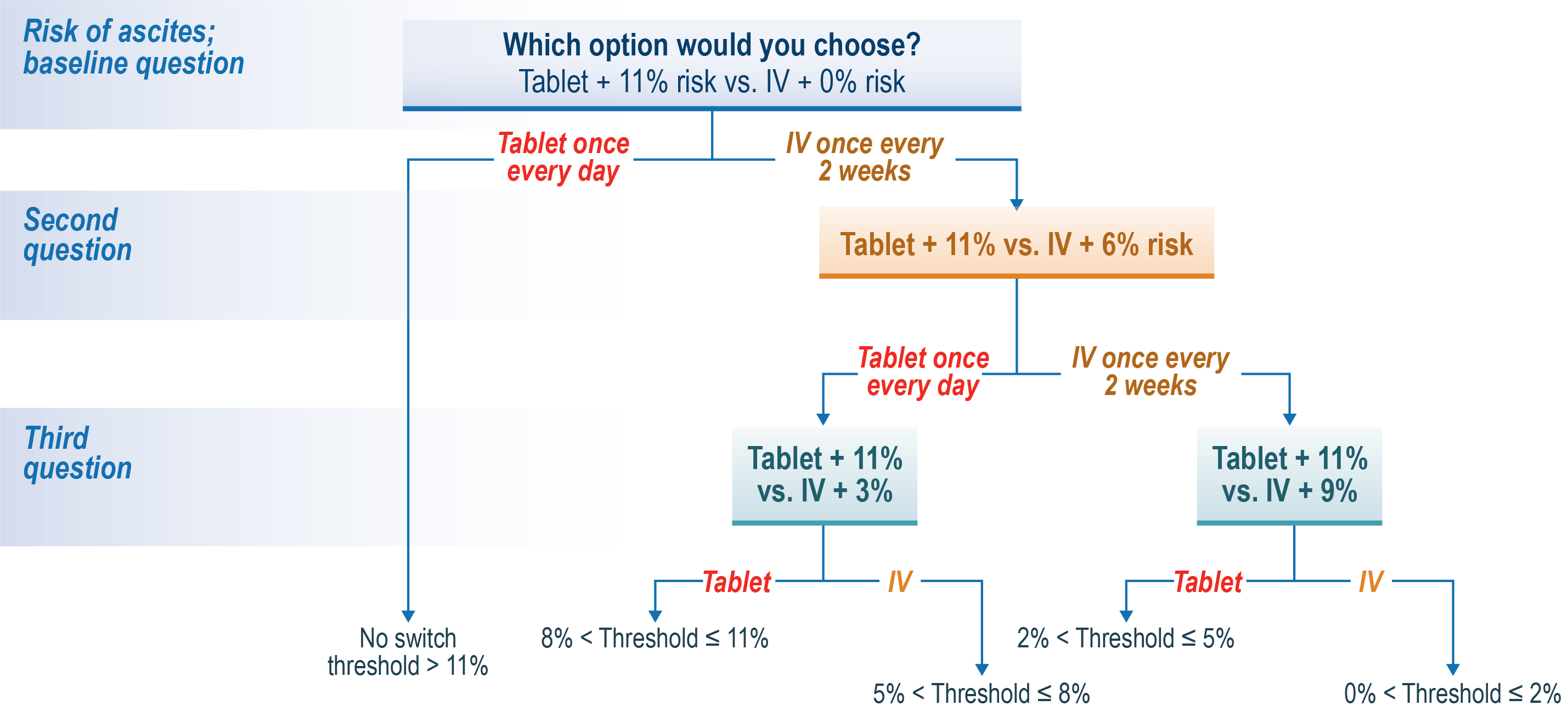


Figure S5. Threshold Question Sequence for Risk of Proteinuria if “Four Tablets” is Initially Preferred


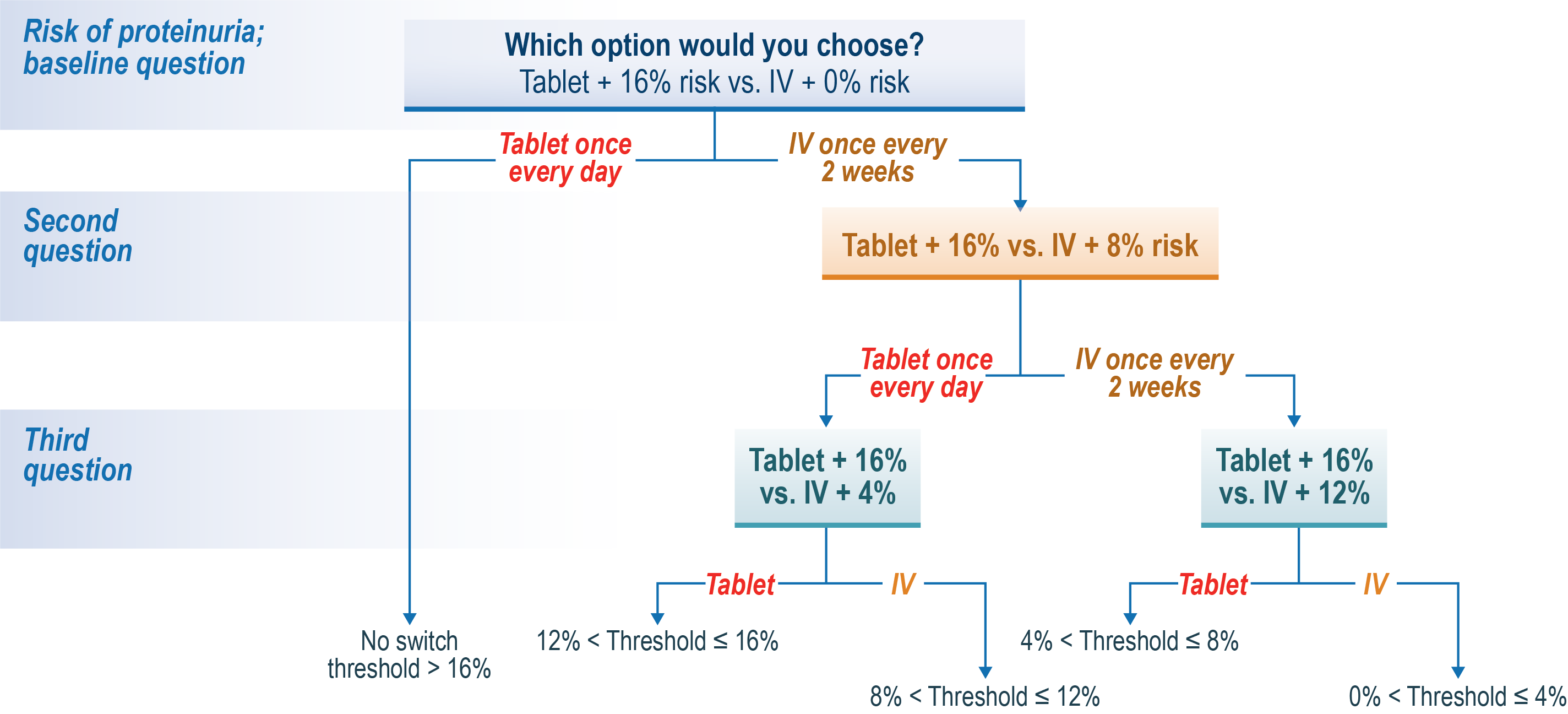


Figure S6. Threshold Question Sequence for Risk of Peripheral Edema if “Four Tablets” is Initially Preferred


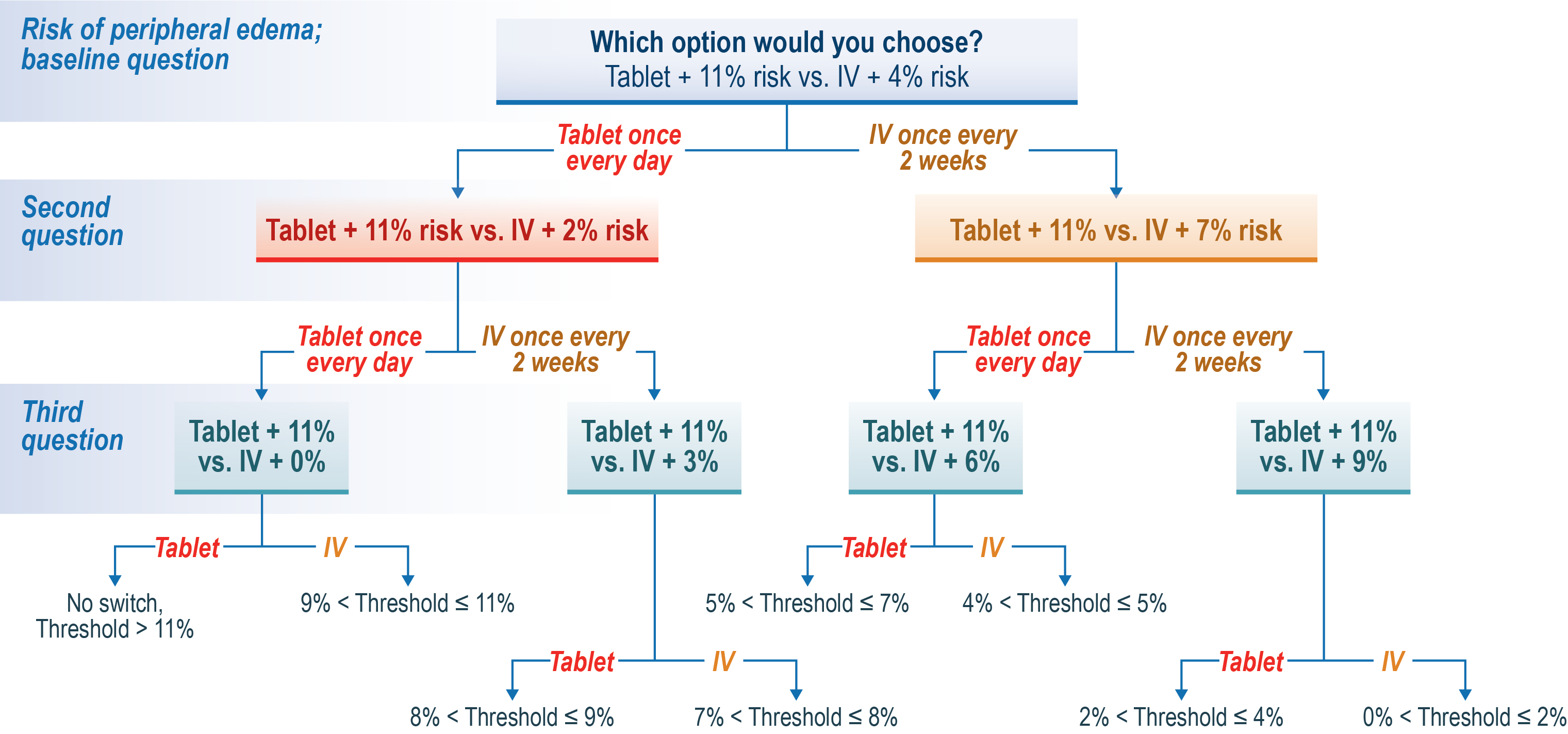


Figure S7. Threshold Question Sequence for Risk of Hypertension if Intravenous Infusion is Initially Preferred


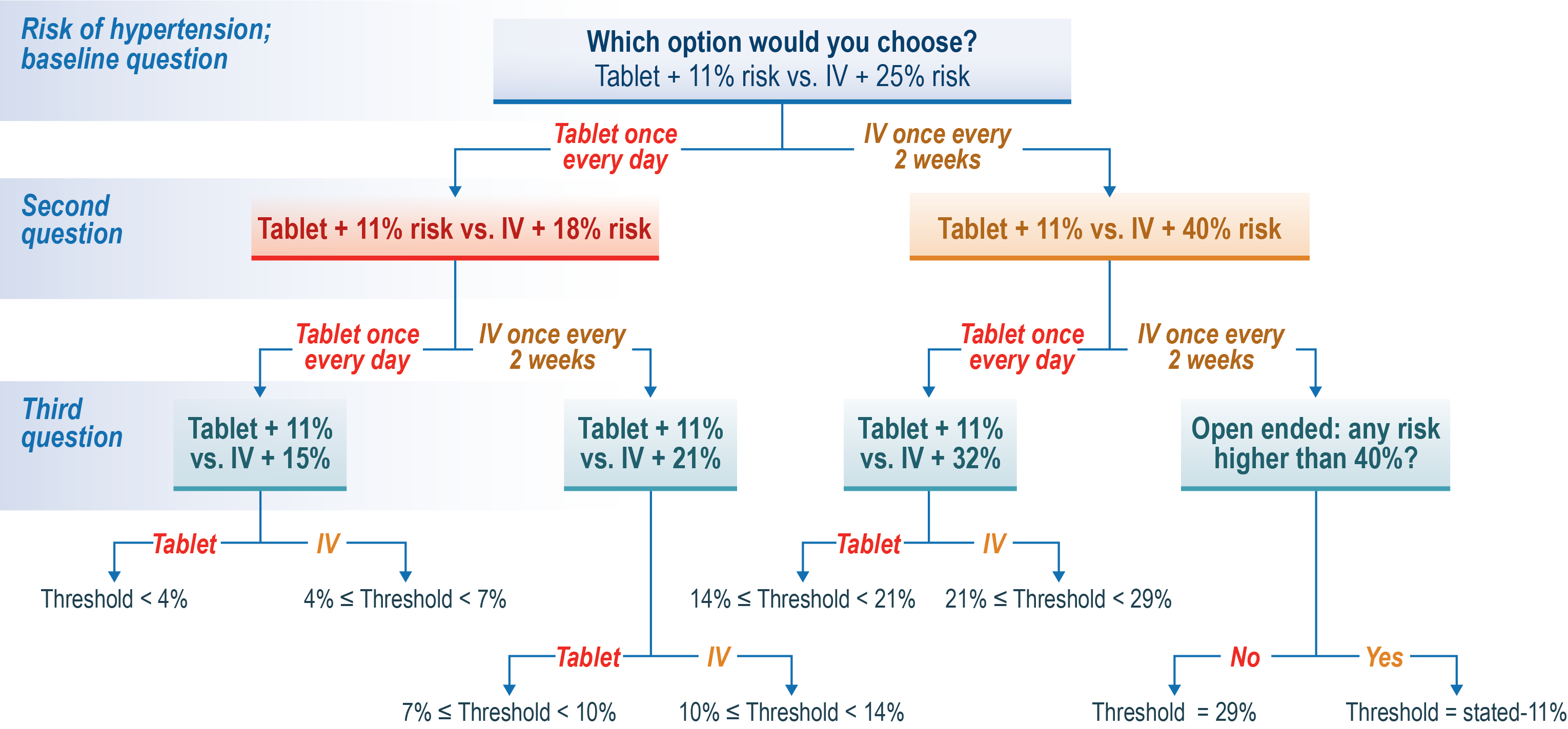


Figure S8. Threshold Question Sequence for Risk of Decreased Appetite if Intravenous Infusion is Initially Preferred


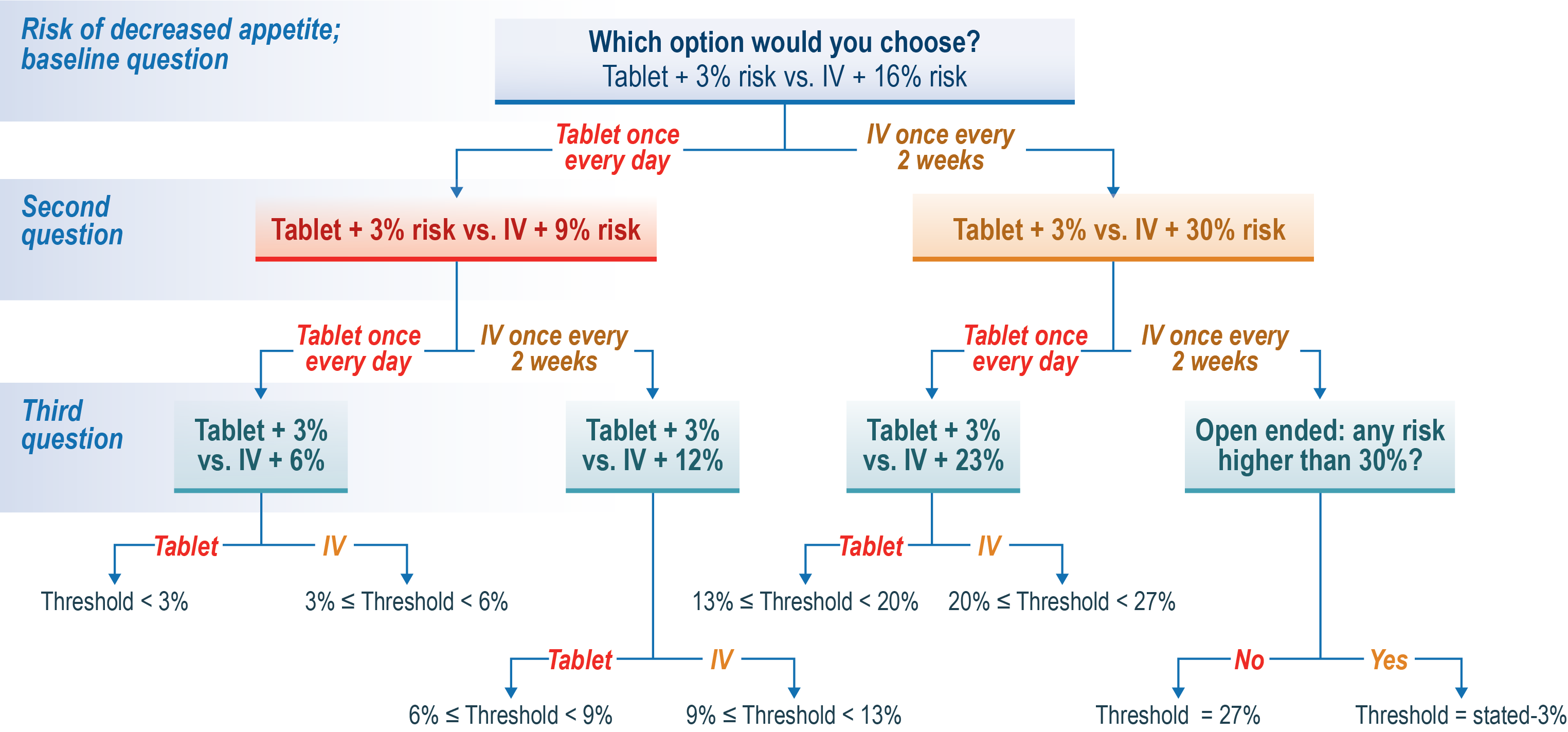


Figure S9. Threshold Question Sequence for Risk of Hand-Foot Skin Reaction if Intravenous Infusion is Initially Preferred


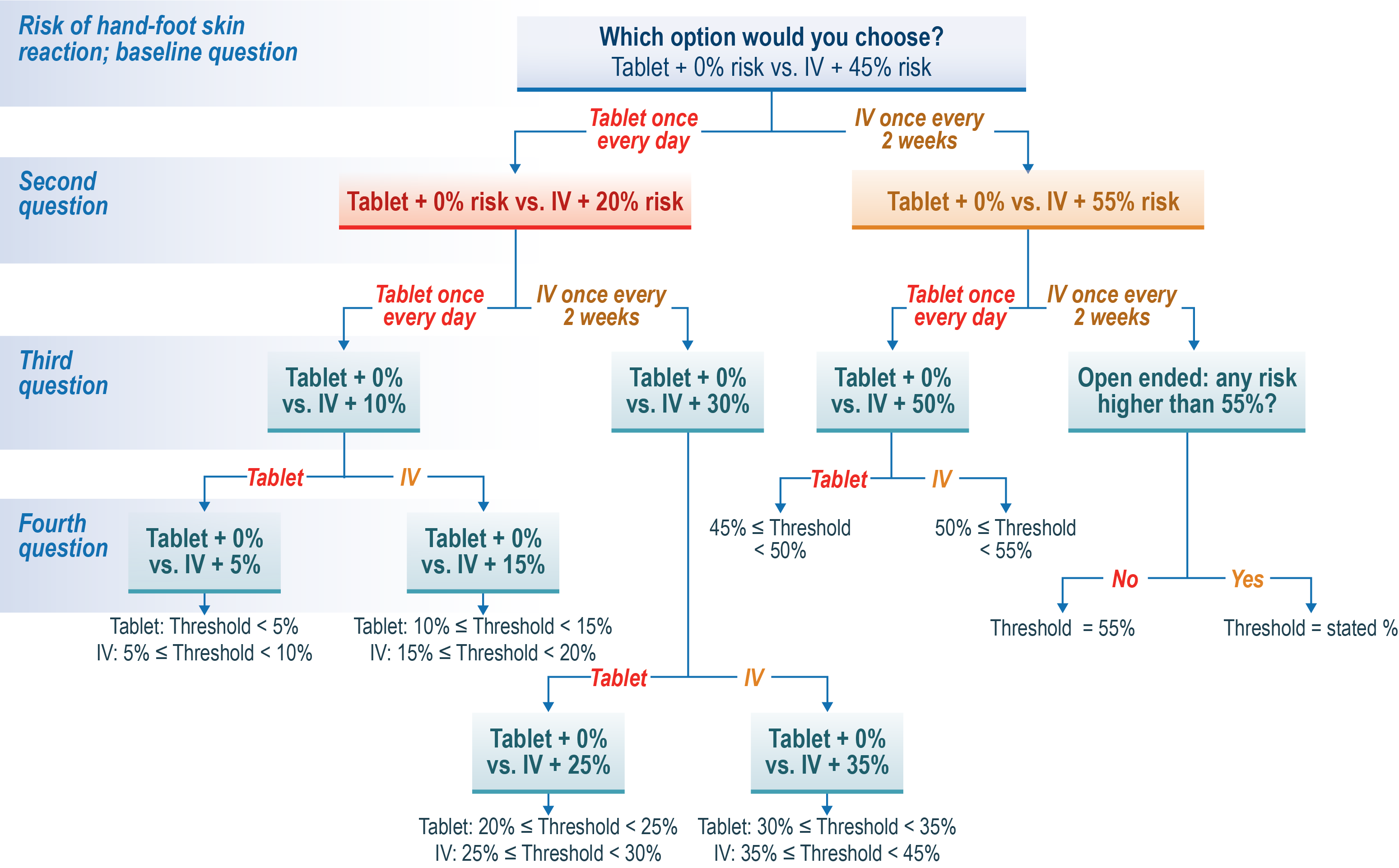


Figure S10. Threshold Question Sequence for Risk of Diarrhea if Intravenous Infusion is Initially Preferred


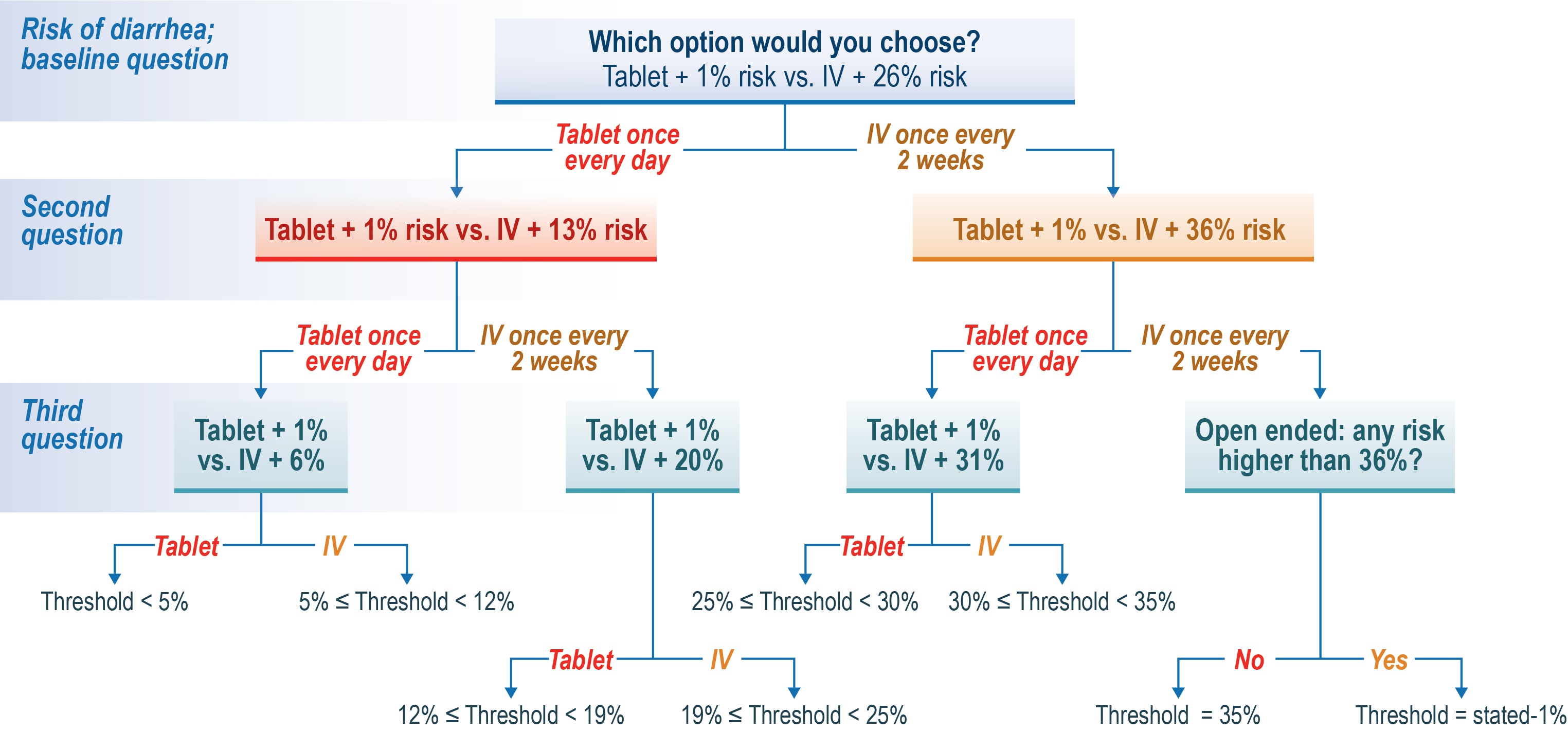


Figure S11. Threshold Question Sequence for Risk of Ascites if Intravenous Infusion is Initially Preferred


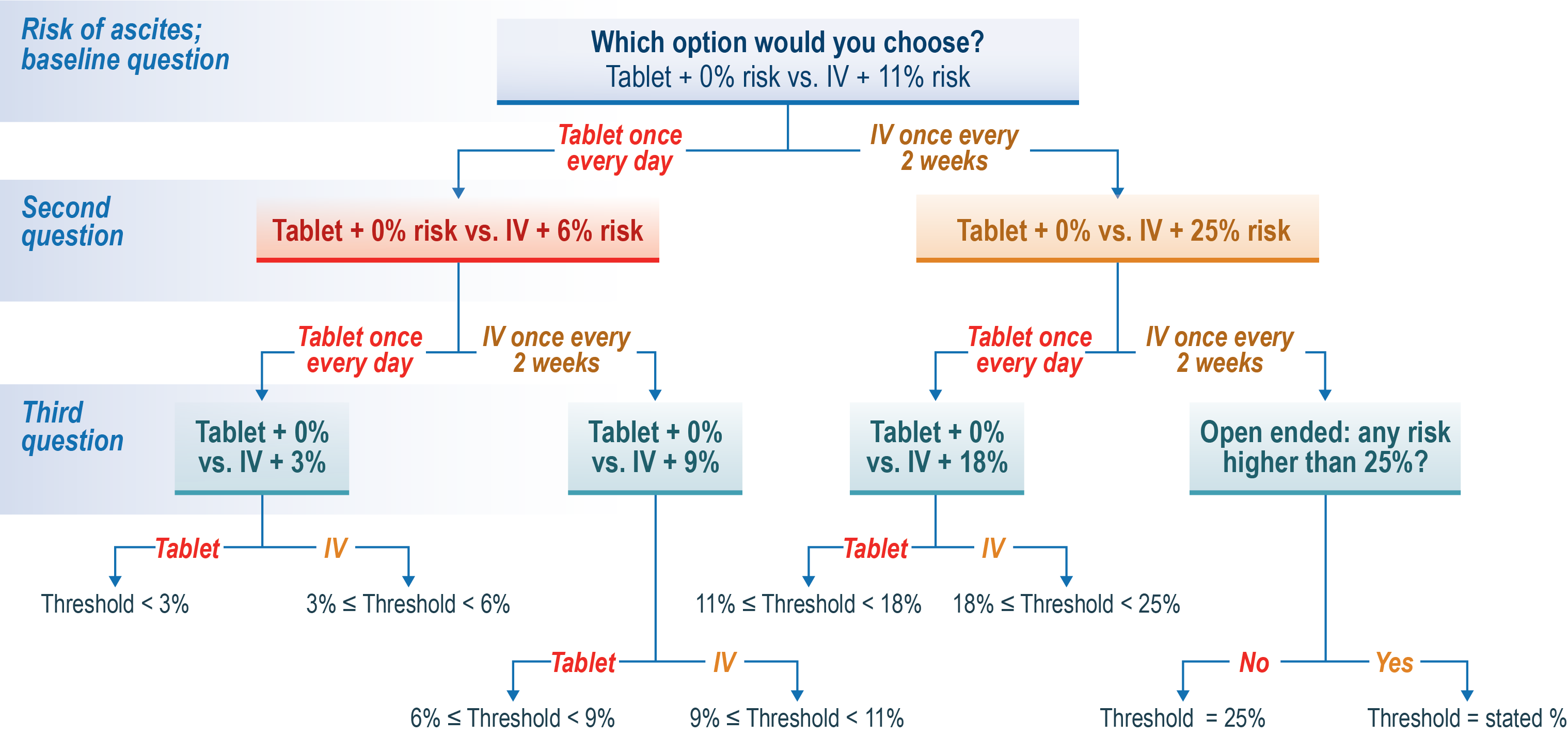


Figure S12. Threshold Question Sequence for Risk of Proteinuria if Intravenous Infusion is Initially Preferred


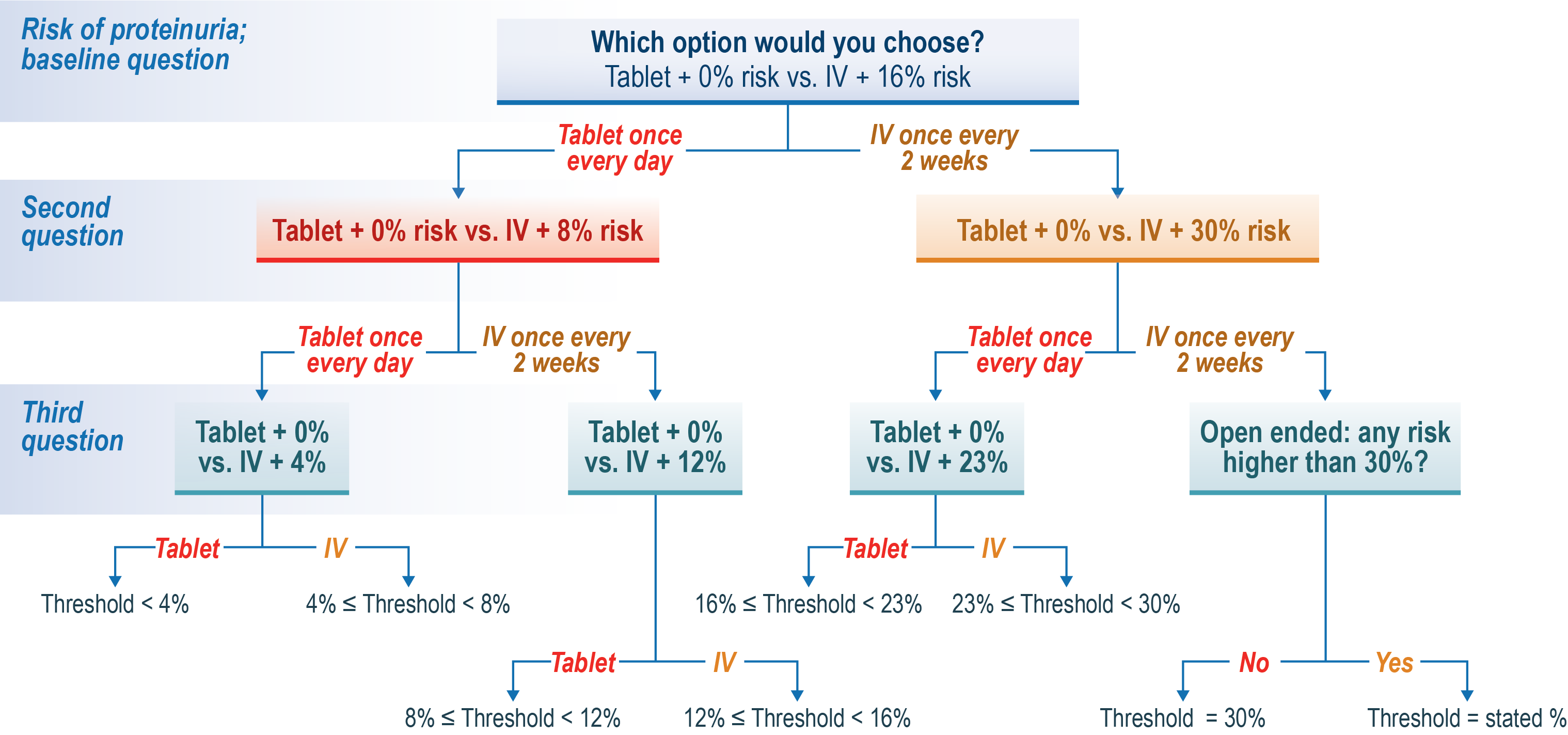


Figure S13. Threshold Question Sequence for Risk of Peripheral Edema if Intravenous Infusion is Initially Preferred


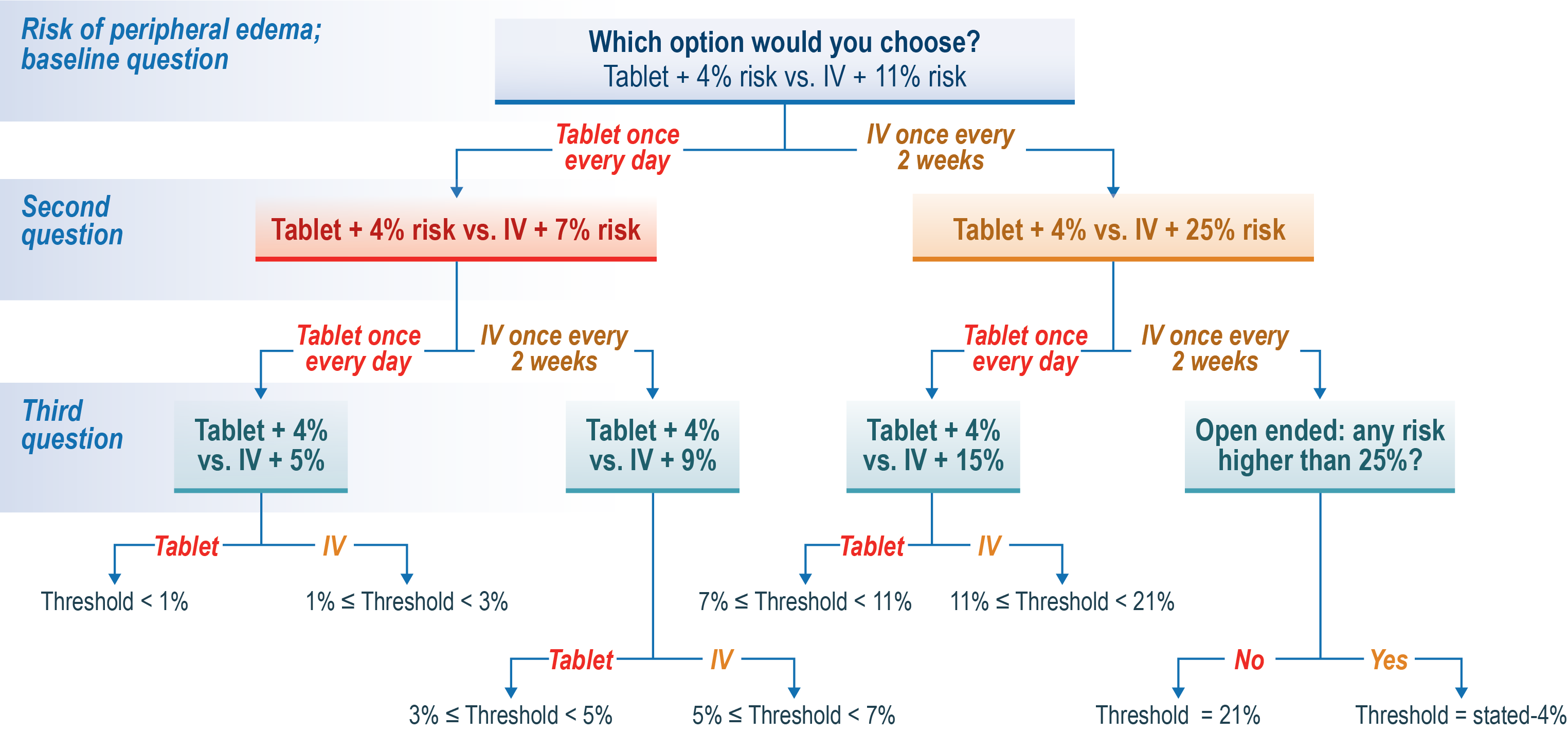


Figure S14. Minimum Reduction in Risk of Hypertension to Switch From Tablets Every Day to Intravenous Infusion Every 2 Weeks


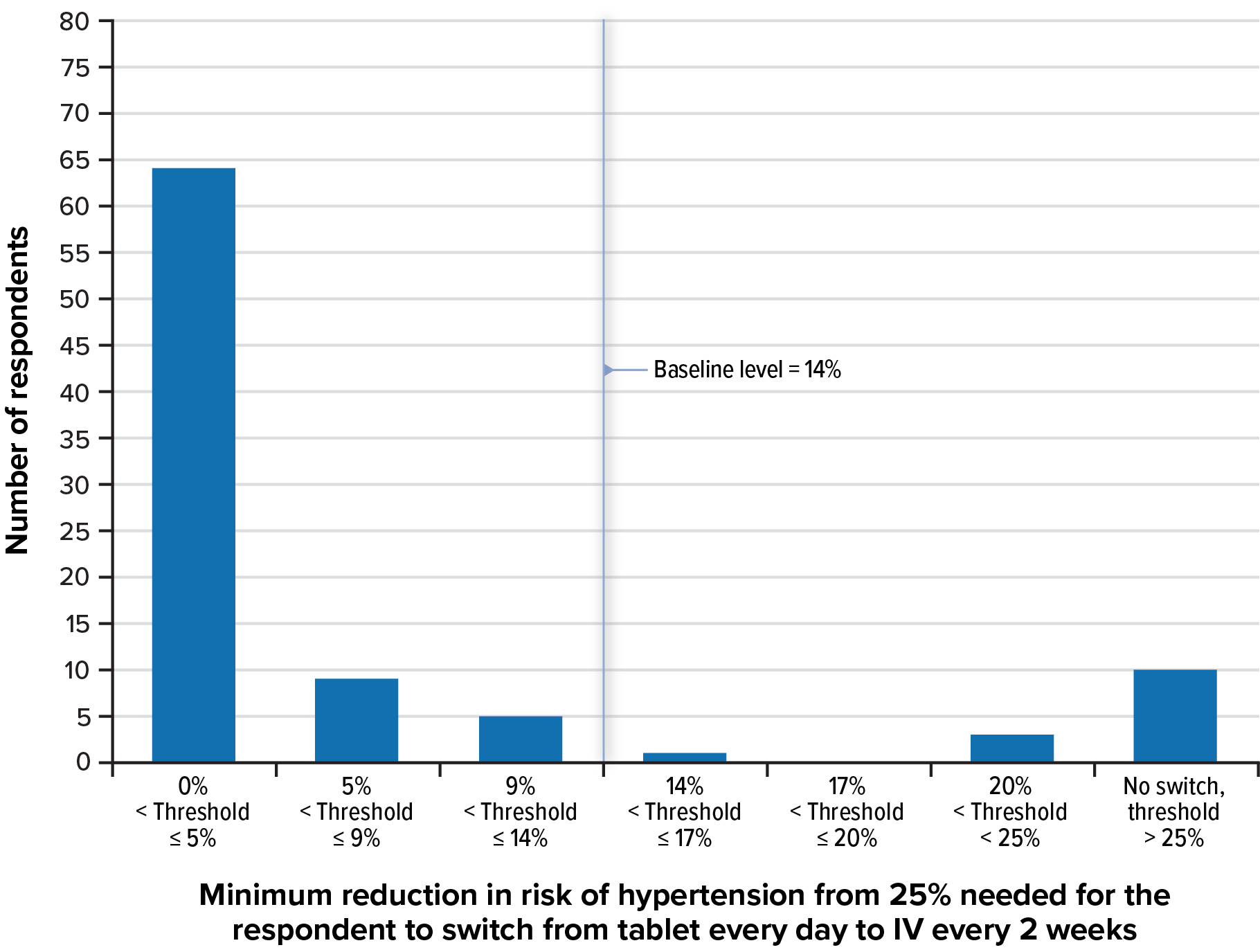


IV = intravenous infusion.

Note: Of the 92 people (61.3% of the full sample) who preferred tablets to IV (all else equal), only 14 (15.2%) still preferred tablets if the risk of hypertension was 14 percentage points higher with the tablets than with the IV. The mean maximum acceptable risk difference was 7.07%, which is less than the 14 percentage-point difference between regorafenib and ramucirumab.

Figure S15. Minimum Reduction in Risk of Decreased Appetite to Switch From Tablets Every Day to Intravenous Infusion Every 2 Weeks


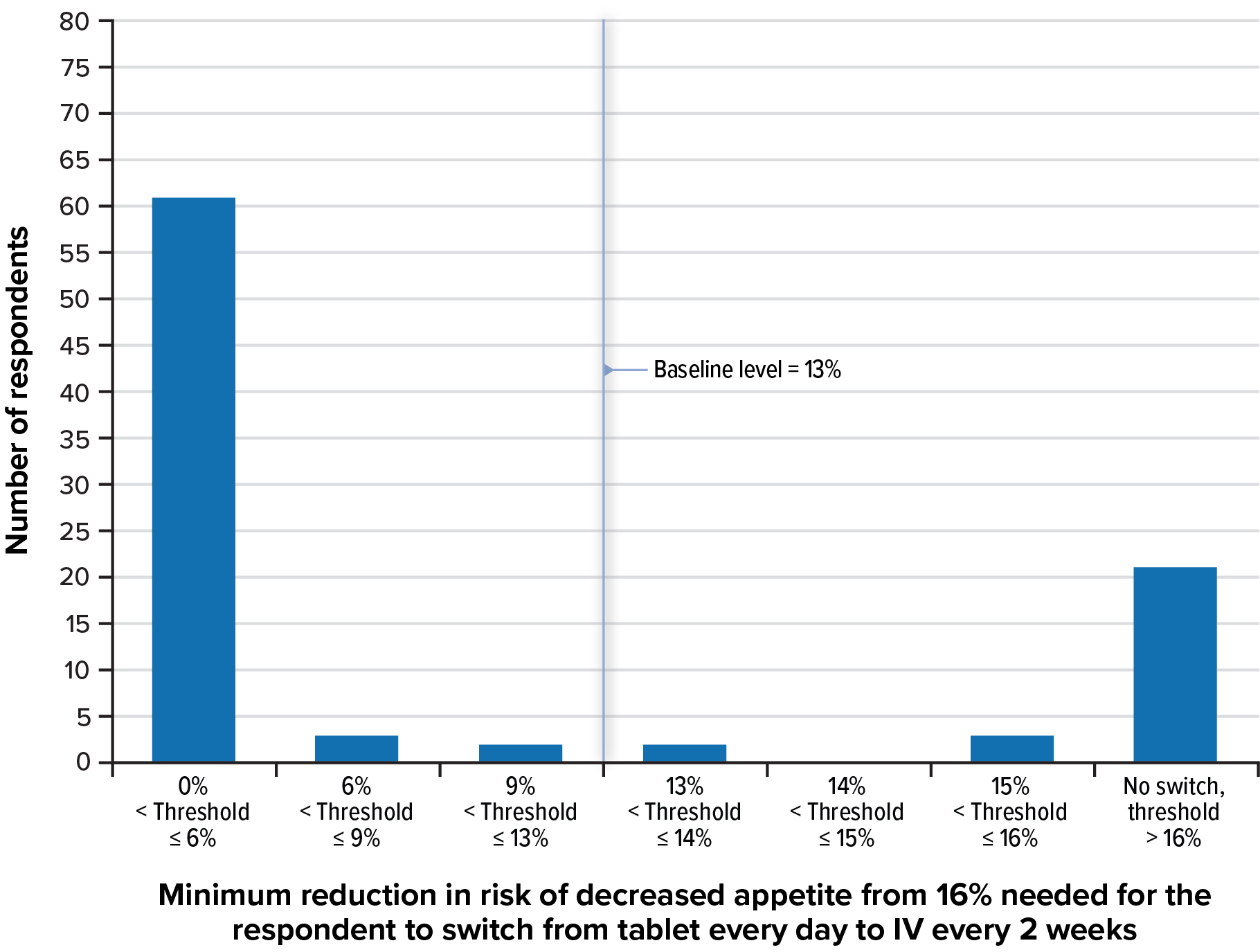


IV = intravenous infusion.

Note: Of the 92 people (61.3% of the full sample) who preferred tablets to IV (all else being equal), only 26 (28.3%) still preferred tablets if the risk of decreased appetite was 13 percentage points higher with the tablets than with the IV. The mean maximum acceptable risk difference was 7.94%, which is less than the 13 percentage- point difference between regorafenib and ramucirumab.

Figure S16. Minimum Reduction in Risk of Hand-Foot Skin Reaction to Switch From Tablets Every Day to Intravenous Infusion Every 2 Weeks


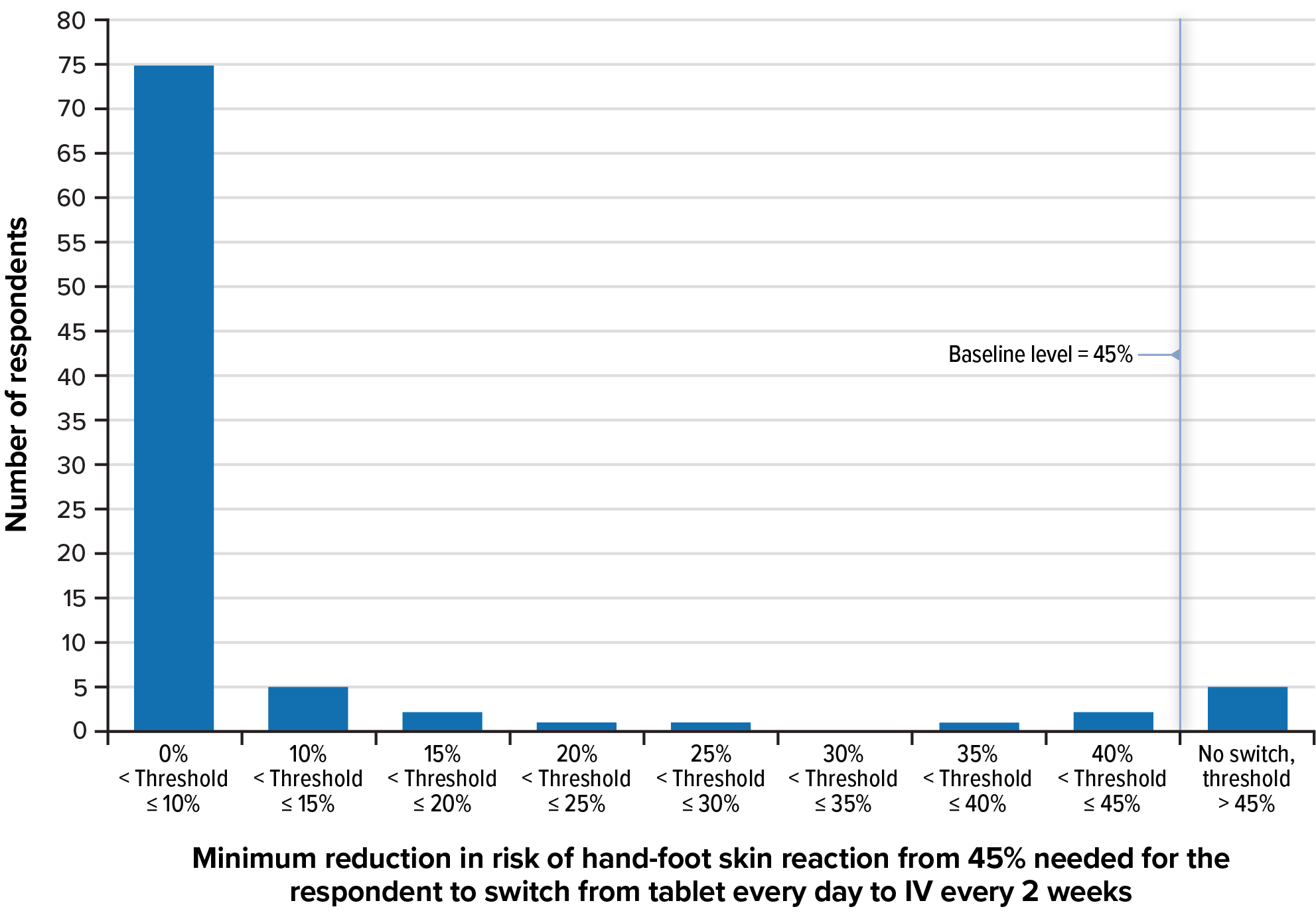


IV = intravenous infusion.

Note: Of the 92 people (61.3% of the full sample) who preferred tablets to IV (all else being equal), only 5 (5.4%) still preferred tablets if the risk of hand-foot skin reaction was 45 percentage points higher with the tablets than with the IV. The mean maximum acceptable risk difference was 9.84%, which is less than the 45 percentage-point difference between regorafenib and ramucirumab.

Figure S17. Minimum Reduction in Risk of Diarrhea to Switch From Tablets Every Day To Intravenous Infusion Every 2 Weeks


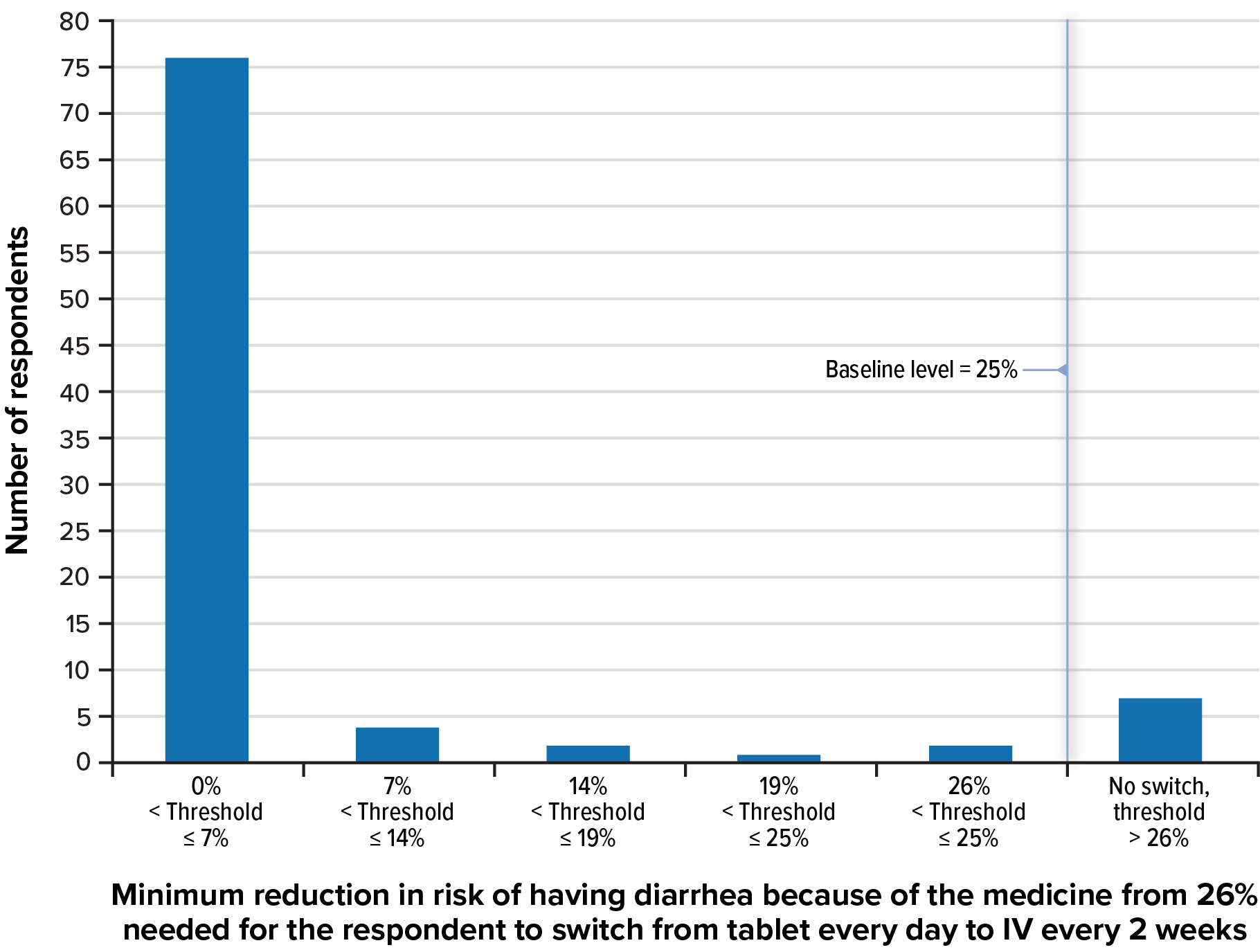


IV = intravenous infusion.

Note: Of the 92 people (61.3% of the full sample) who preferred tablets to IV (all else being equal), only 9 (9.8%) still preferred tablets if the risk of diarrhea was 25 percentage points higher with the tablets than with the IV. The mean maximum acceptable risk difference was 6.83%, which is less than the 25 percentage-point difference between regorafenib and ramucirumab.

Figure S18. Minimum Reduction in Risk of Ascites to Switch From Tablets Every Day to Intravenous Infusion Every 2 Weeks


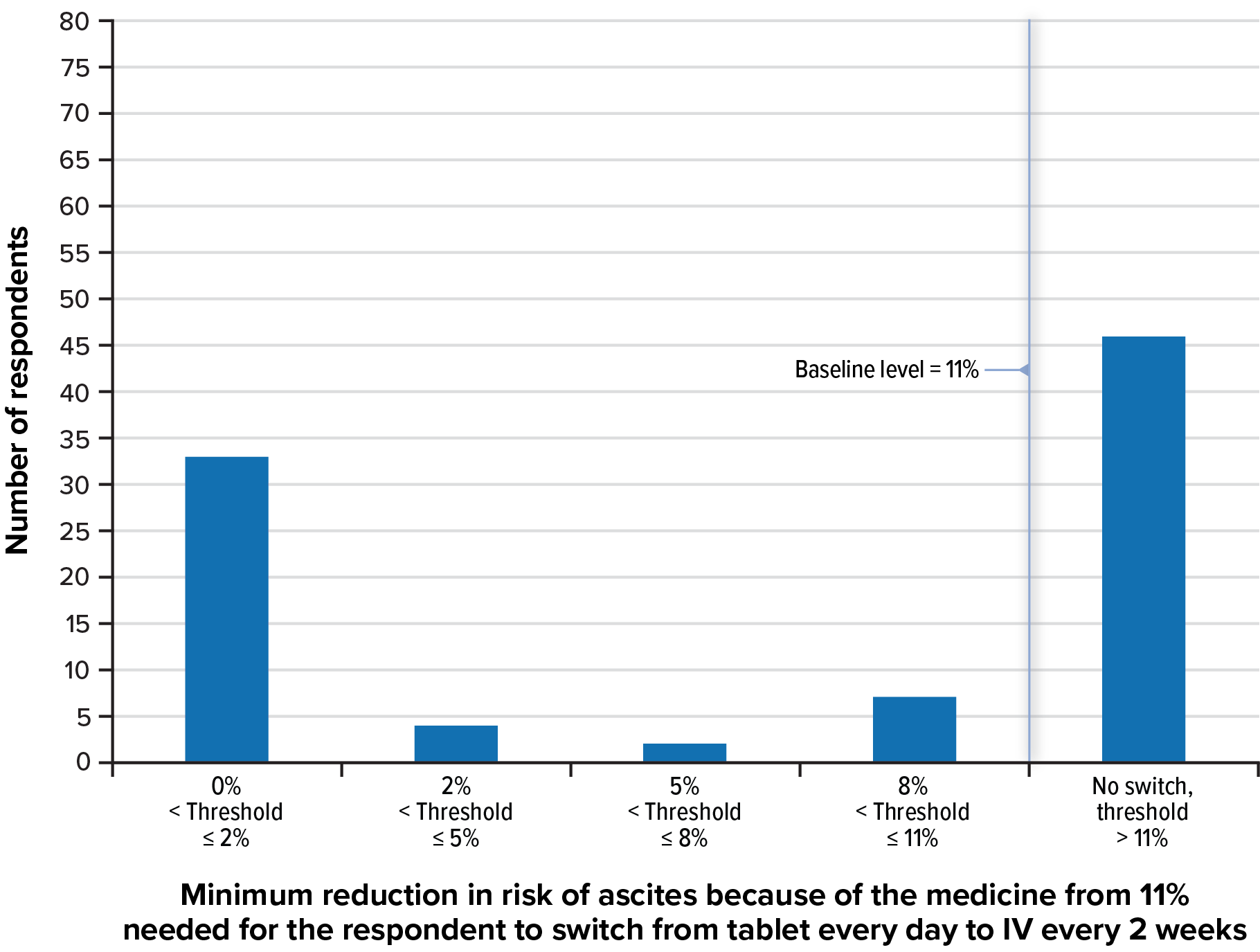


IV = intravenous infusion.

Note: Of the 92 people (61.3% of the full sample) who preferred tablets to IV (all else being equal), 46 (50.0%) still preferred tablets if the risk of ascites was 11 percentage points higher with the tablets than with the IV. The mean maximum acceptable risk difference was 10.07%, which is less than the 11 percentage-point difference between regorafenib and ramucirumab.

Figure S19. Minimum Reduction in Risk of Proteinuria to Switch From Tablets Every Day to Intravenous Infusion Every 2 Weeks


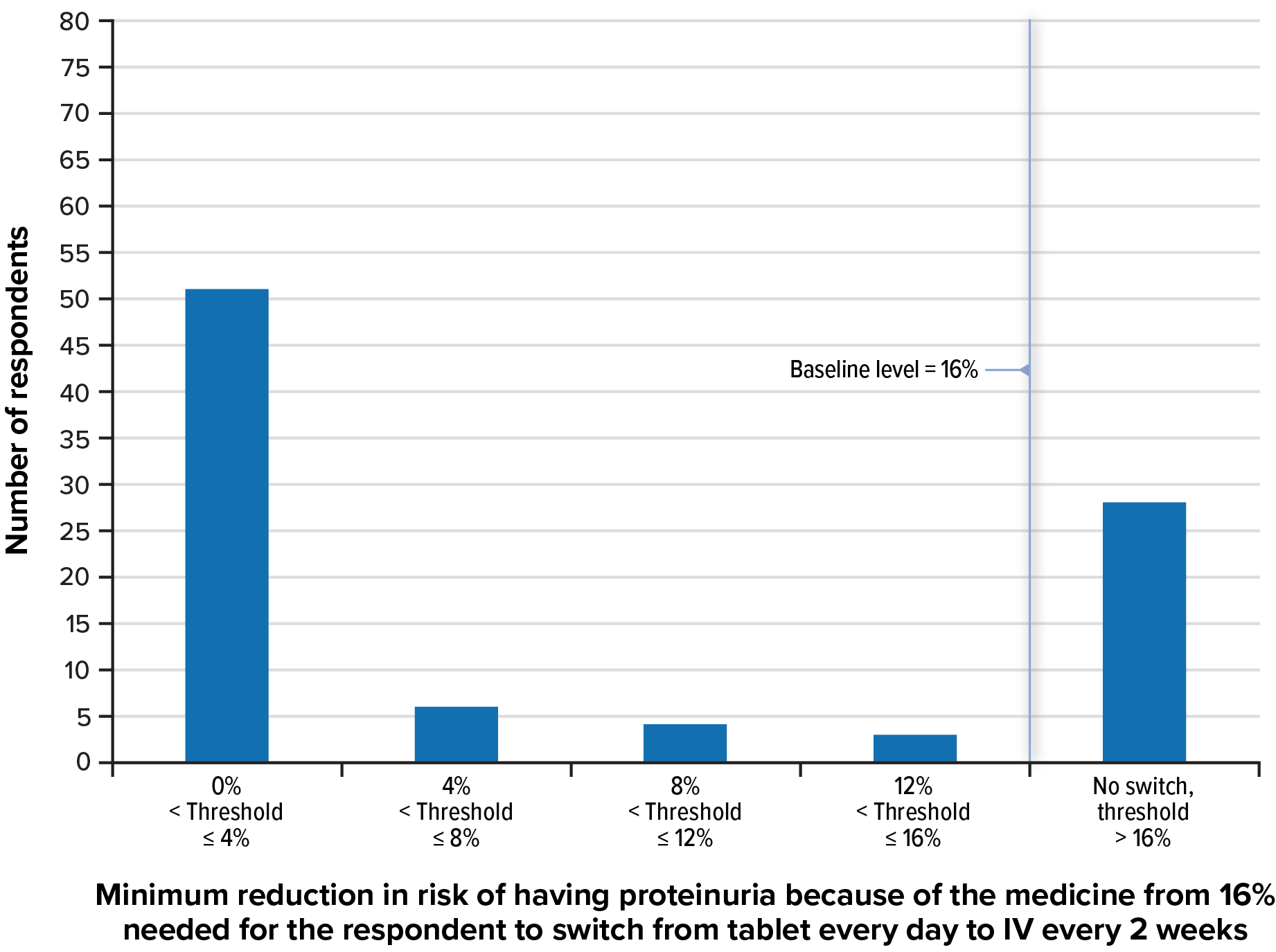


IV = intravenous infusion.

Note: Of the 92 people (61.3% of the full sample) who preferred tablets to IV (all else being equal), only 28 (30.4%) still preferred tablets if the risk of proteinuria was 16 percentage points higher with the tablets than with the IV. The mean maximum acceptable risk difference was 8.83%, which is less than the 16 percentage-point difference between regorafenib and ramucirumab.

Figure S20. Minimum Reduction in Risk of Peripheral Edema to Switch From Tablets Every Day to Intravenous Infusion Every 2 Weeks


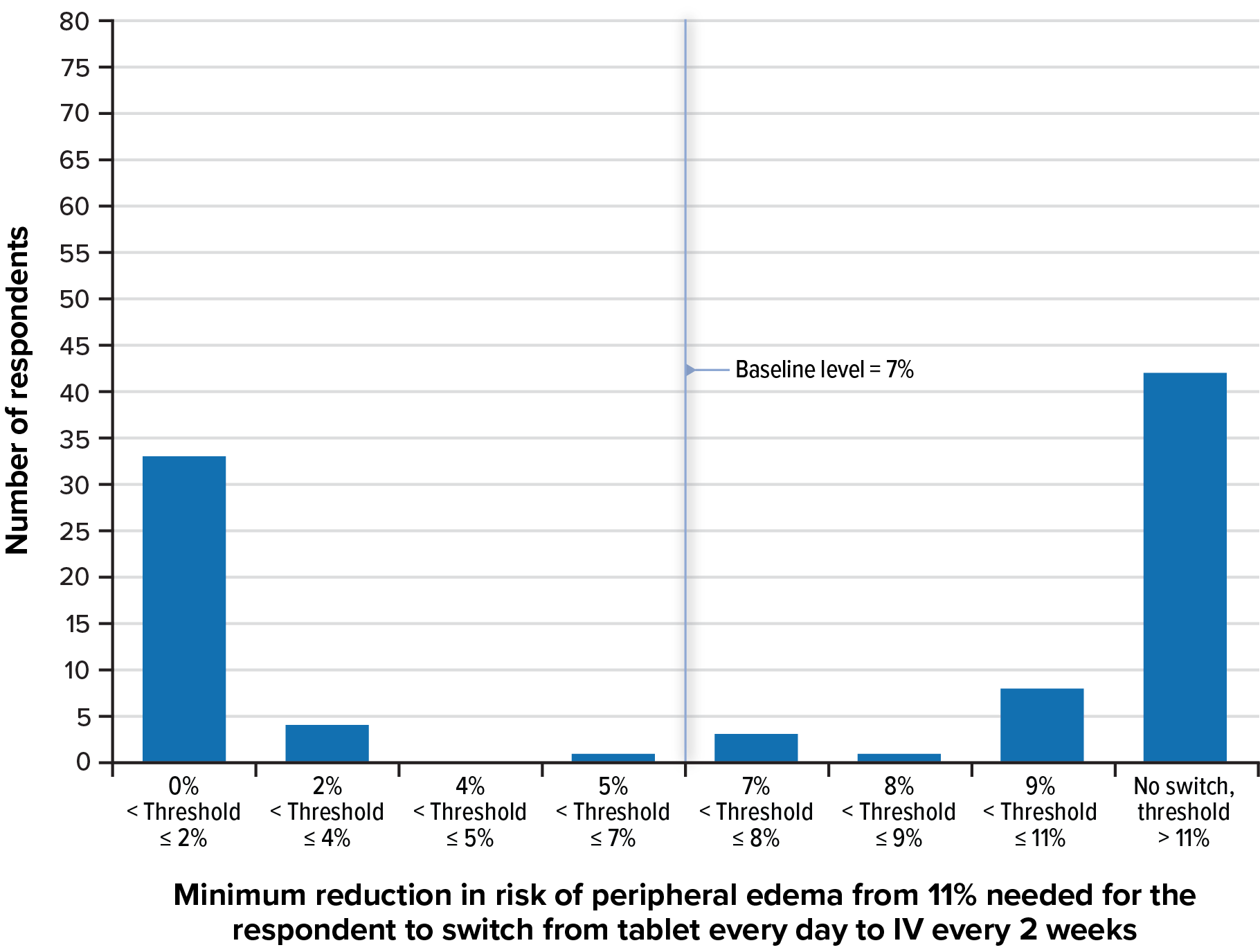


IV = intravenous infusion.

Note: Of the 92 people (61.3% of the full sample) who preferred tablets to IV (all else equal), 54 (58.7%) still preferred tablets if the risk of peripheral edema was 7 percentage points higher with the tablets than with the IV. The mean maximum acceptable risk difference was 9.36%, which is more than the 7 percentage-point difference between regorafenib and ramucirumab.

Figure S21. Maximum Increase in Risk of Hypertension to Keep Intravenous Infusion Every 2 Weeks Instead of Switching to Tablets Every Day


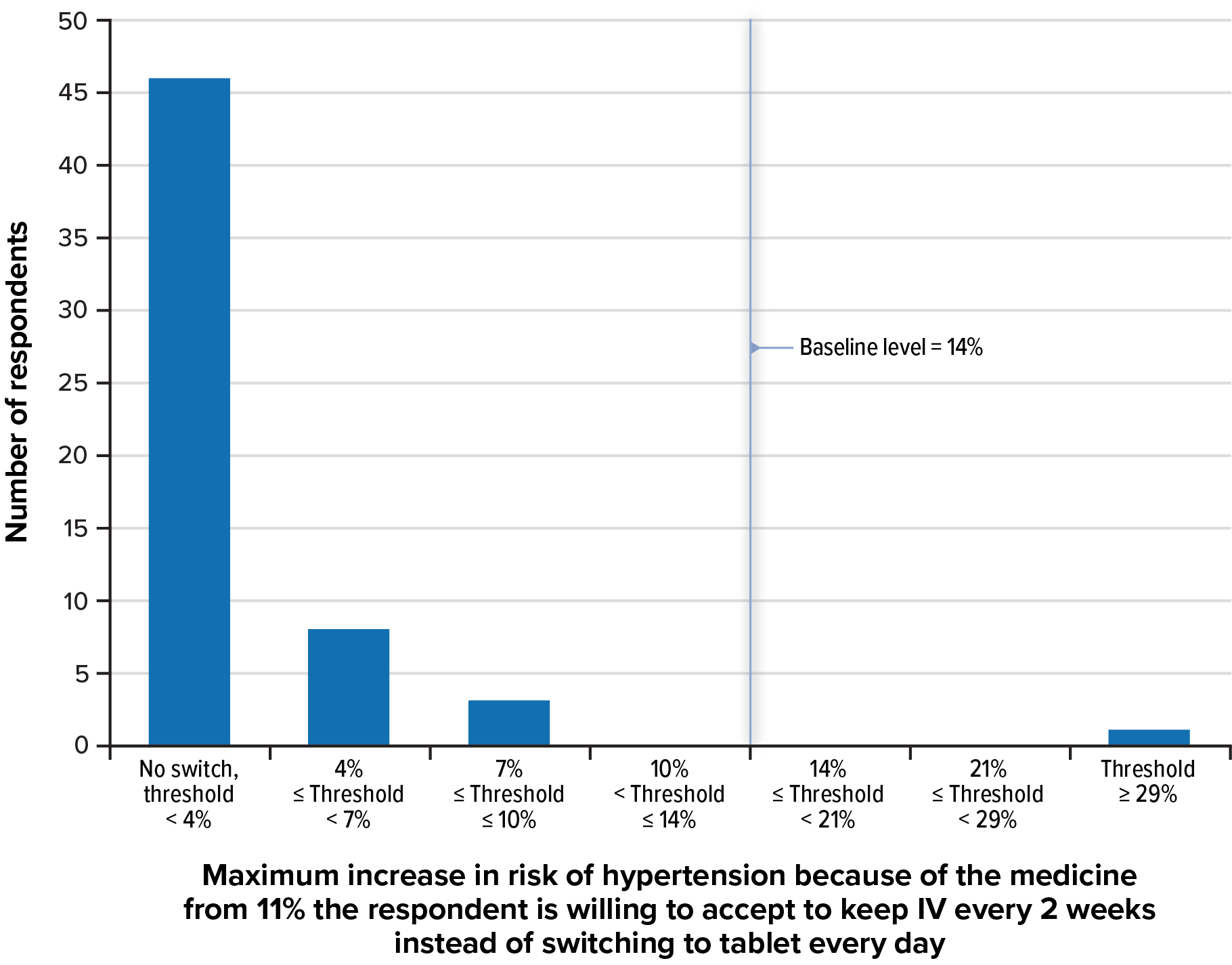


IV = intravenous infusion.

Note: Of the 58 people (38.7% of the full sample) who preferred IV to tablets (all else equal), only 1 (1.7%) still preferred IV if the risk of hypertension was 14 percentage points higher with the IV than with the tablets. The mean maximum acceptable risk difference was 3.36%, which is less than the 14 percentage-point difference between regorafenib and ramucirumab.

Figure S22. Maximum Increase in Risk of Lower Appetite to Keep Intravenous Infusion Every 2 Weeks Instead of Switching to Tablets Every Day


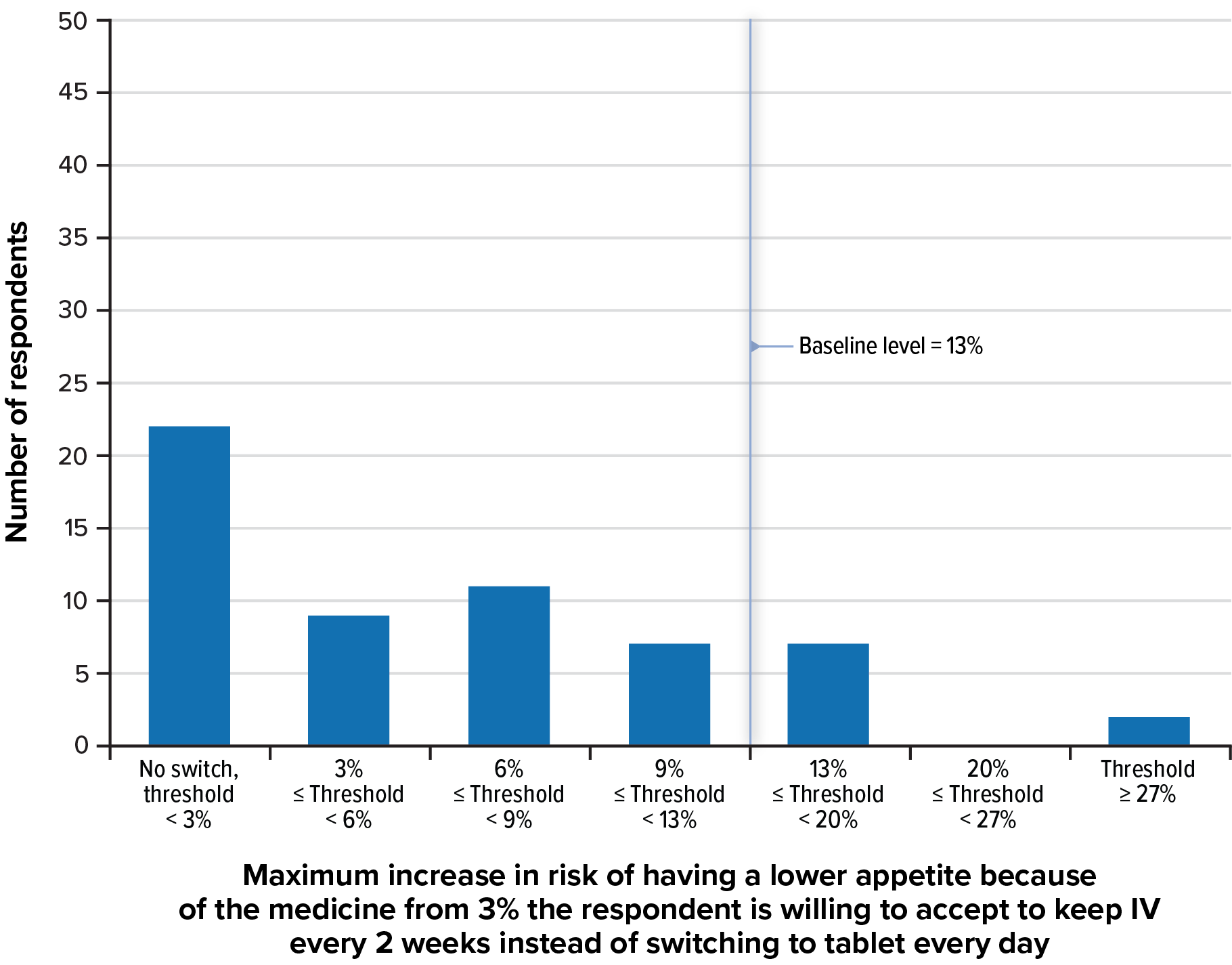


IV = intravenous infusion.

Note: Of the 58 people (38.7% of the full sample) who preferred IV to tablets (all else being equal), only 9 (15.5%) still preferred IV if the risk of lower appetite was 13 percentage points higher with the IV than with the tablets. The mean maximum acceptable risk difference was 6.92%, which is less than the 13 percentage-point difference between regorafenib and ramucirumab.

Figure S23. Maximum Increase in Risk of Hand-foot Reaction to Keep Intravenous Infusion Every 2 Weeks Instead of Switching to Tablets Every Day


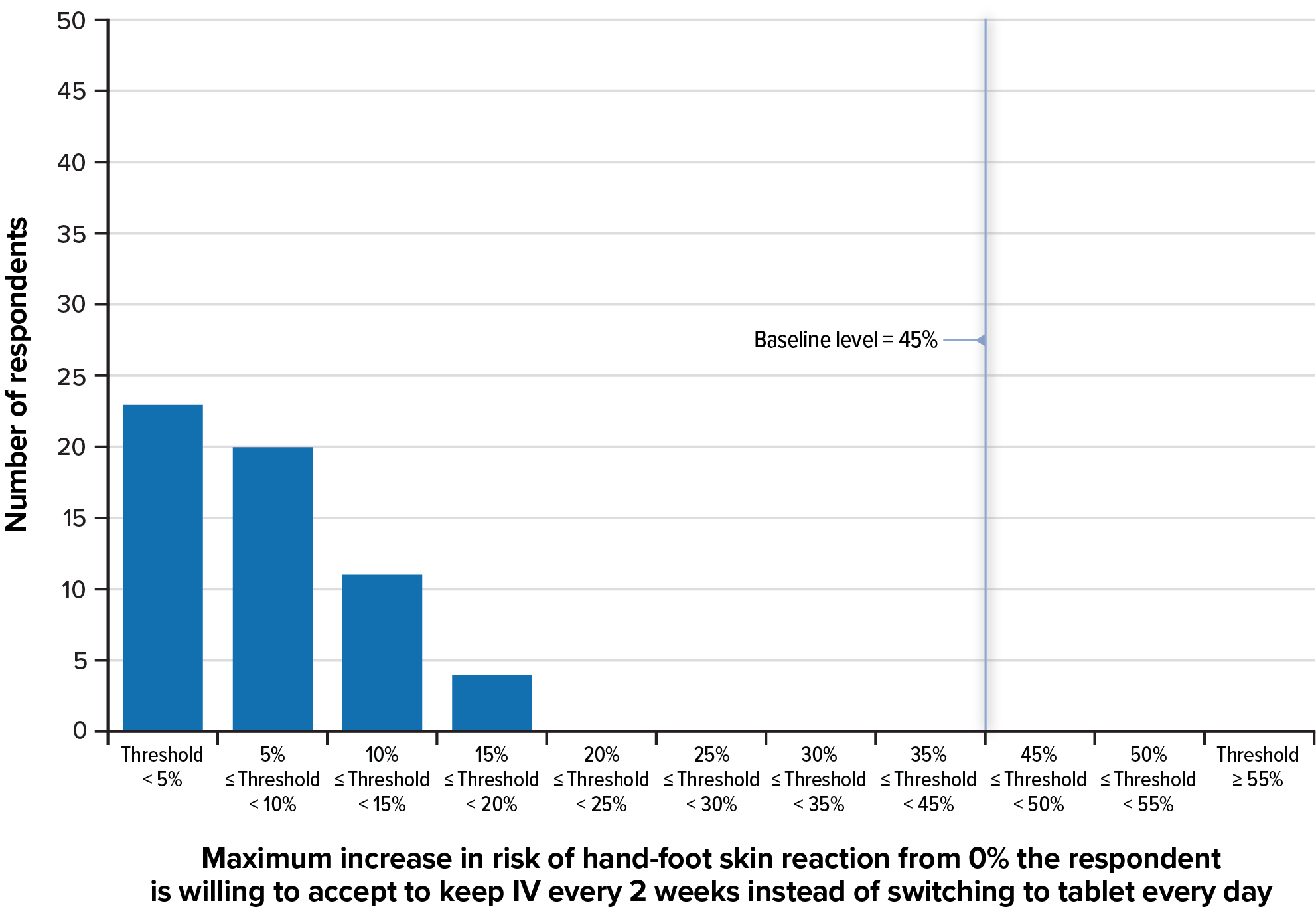


IV = intravenous infusion.

Note: Of the 58 people (38.7% of the full sample) who preferred IV to tablets (all else equal), 0 preferred IV if the risk of hand-foot reaction was 45 percentage points higher with the IV than with the tablets. The mean maximum acceptable risk difference was 7.16%, which is less than the 45 percentage-point difference between regorafenib and ramucirumab.

Figure S24. Maximum Increase in Risk of Diarrhea to Keep Intravenous Infusion Every 2 Weeks Instead of Switching to Tablets Every Day


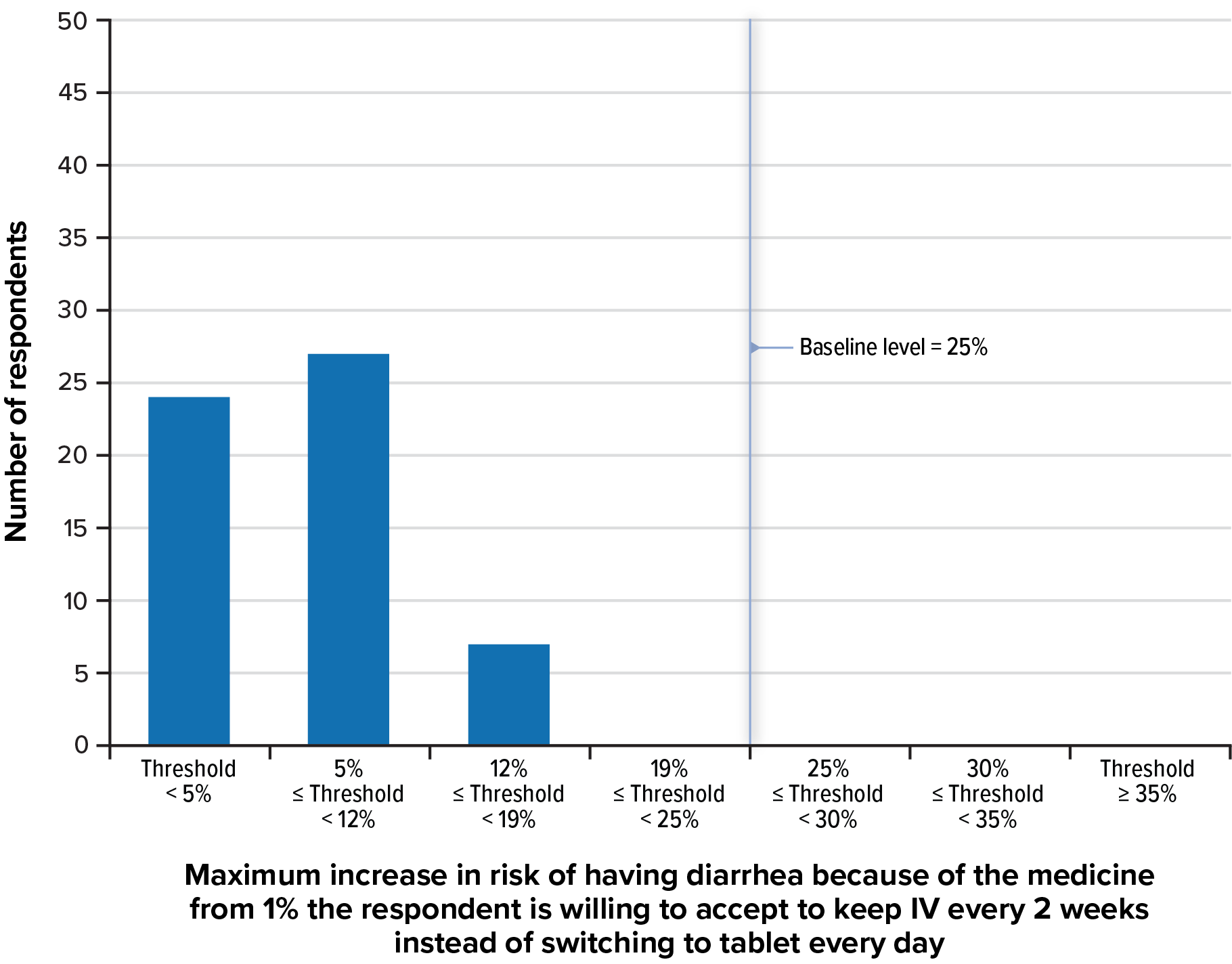


IV = intravenous infusion.

Note: Of the 58 people (38.7% of the full sample) who preferred IV to tablets (all else being equal), 0 preferred IV if the risk of diarrhea was 25 percentage points higher with the IV than with the tablets. The mean maximum acceptable risk difference was 6.66%, which is less than the 25 percentage-point difference between regorafenib and ramucirumab.

Figure S25. Maximum Increase in Risk of Ascites to Keep Intravenous Infusion Every 2 Weeks Instead of Switching to Tablets Every Day


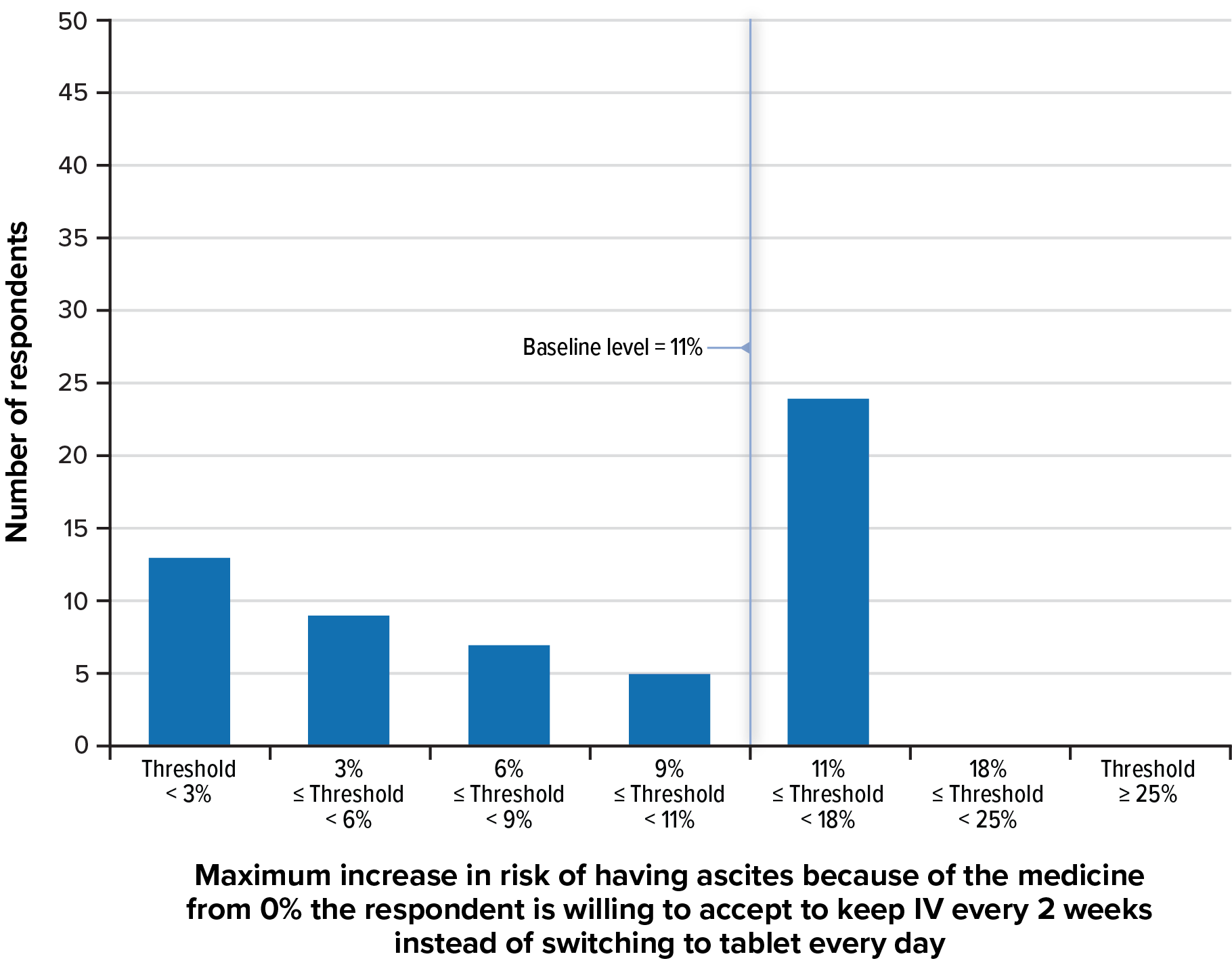


IV = intravenous infusion.

Note: Of the 58 people (38.7% of the full sample) who preferred IV to tablets (all else equal), 24 (41.4%) preferred IV if the risk of ascites was 11 percentage points higher with the IV than with the tablets. The mean maximum acceptable risk difference was 8.51% which is less than the 11 percentage-point difference between regorafenib and ramucirumab.

Figure S26. Maximum Increase in Risk of Proteinuria to Keep Intravenous Infusion Every 2 Weeks Instead of Switching to Tablets Every Day


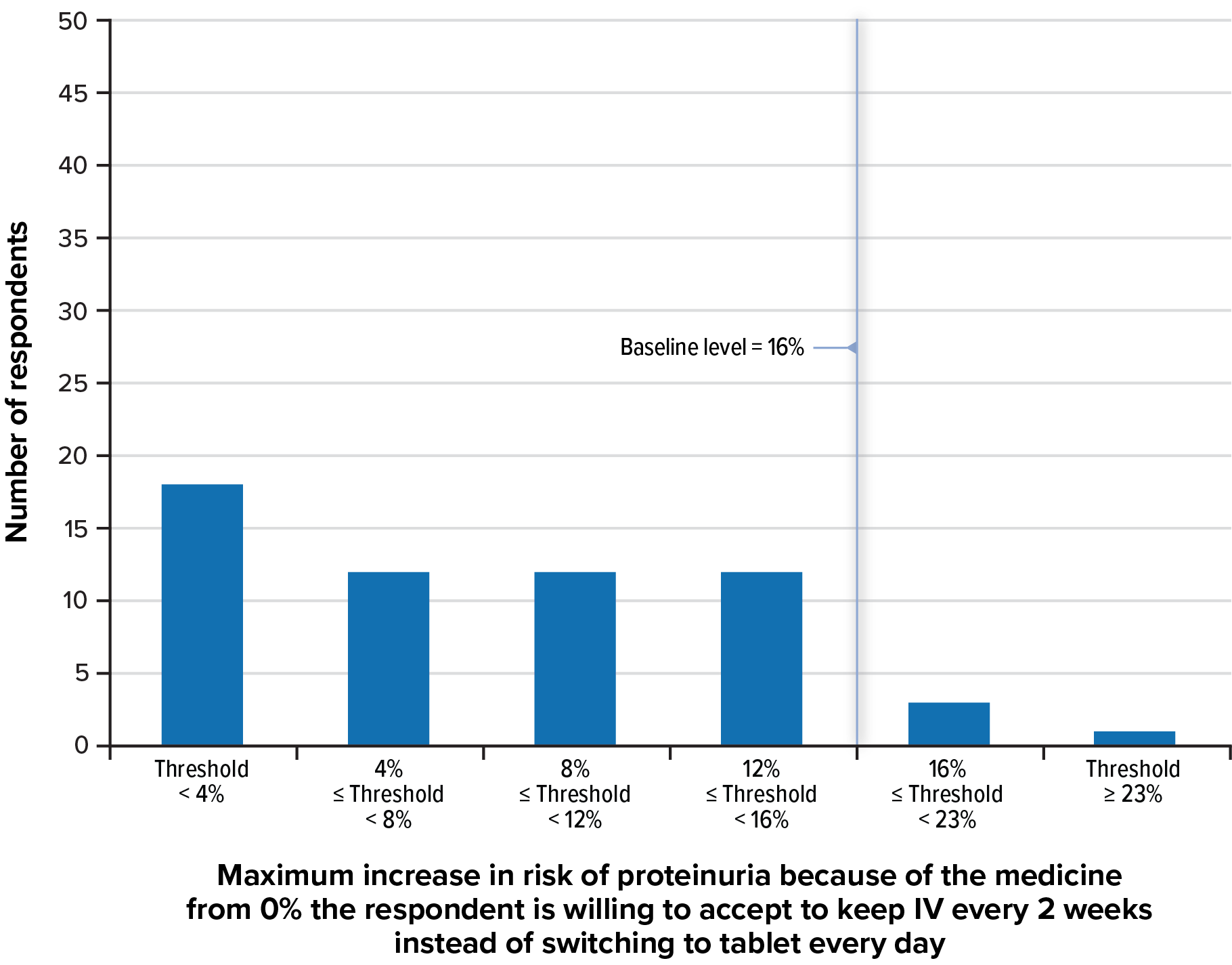


IV = intravenous infusion.

Note: Of the 58 people (38.7% of the full sample) who preferred IV to tablets (all else being equal), only 4 (6.9%) preferred IV if the risk of proteinuria was 16 percentage points higher with the IV than with the tablets. The mean maximum acceptable risk difference was 8.35%, which is less than the 16 percentage-point difference between regorafenib and ramucirumab.

Figure S27. Maximum Increase in Risk of Peripheral Edema to Keep Intravenous Infusion Every 2 Weeks Instead of Switching to Tablets Every Day


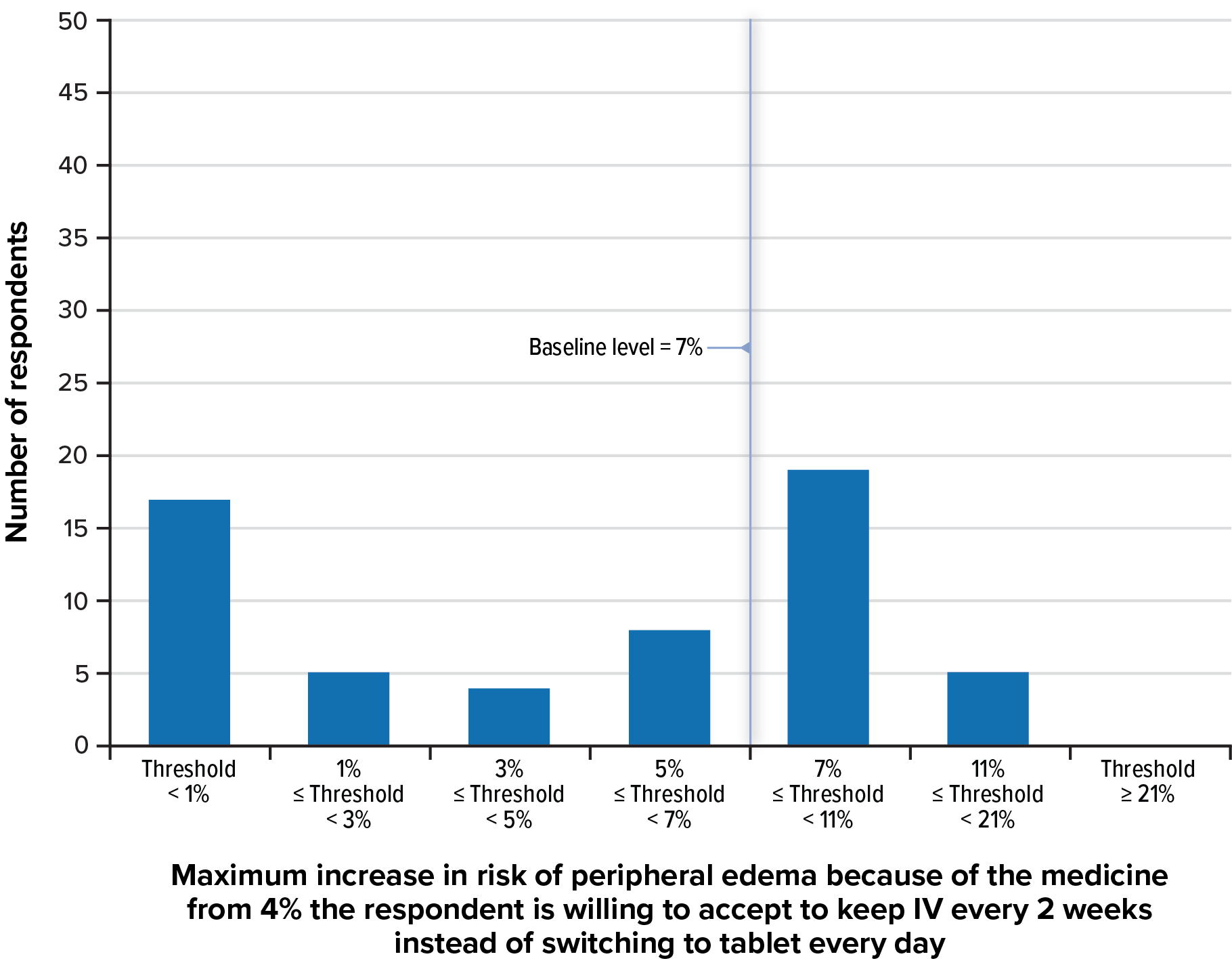


IV = intravenous infusion.

Note: Of the 58 people (38.7% of the full sample) who preferred IV to tablets (all else being equal), 24 (41.4%) preferred IV if the risk of peripheral edema was 7 percentage points higher with the IV than with the tablets. The mean maximum acceptable risk difference was 5.41%, which is less than the 7 percentage-point difference between regorafenib and ramucirumab.
